# Supplementary material for: Rational design of hairpin RNA excited states reveals multi-step transitions
Source: Nat Commun. 2022 Mar 21;13:1523. doi: 10.1038/s41467-022-29194-8 (PMC8938425; doi:10.1038/s41467-022-29194-8)
Supplement: Supplementary file 1 — Supplementary Information [file 41467_2022_29194_MOESM1_ESM.pdf]

Supplementary Information

Rational Design of Hairpin RNA Excited States Reveals Multi-step Transitions

Ge Han, Yi Xue*

School of Life Sciences; Tsinghua-Peking Joint Center for Life Sciences; Beijing Advanced Innovation Center for Structural Biology, Tsinghua University, Beijing, 100084, China

Table of Contents

TABLE OF CONTENTS 1

SUPPLEMENTARY METHODS 2

DESIGN PROTOCOL OF RNA CONSTRUCTS 2
ACCELERATED MOLECULAR DYNAMICS (AMD) SIMULATIONS 4
KINETIC SIMULATIONS 5

SUPPLEMENTARY FIGURES 6

SUPPLEMENTARY FIGURE 1 6
SUPPLEMENTARY FIGURE 2 7
SUPPLEMENTARY FIGURE 3 8
SUPPLEMENTARY FIGURE 4 9
SUPPLEMENTARY FIGURE 5 12
SUPPLEMENTARY FIGURE 6 13
SUPPLEMENTARY FIGURE 7 16
SUPPLEMENTARY FIGURE 8 17
SUPPLEMENTARY FIGURE 9 18
SUPPLEMENTARY FIGURE 10 19
SUPPLEMENTARY FIGURE 11 21
SUPPLEMENTARY FIGURE 12 22
SUPPLEMENTARY FIGURE 13 24
SUPPLEMENTARY FIGURE 14 25
SUPPLEMENTARY FIGURE 15 26
SUPPLEMENTARY FIGURE 16 27
SUPPLEMENTARY FIGURE 17 28
SUPPLEMENTARY FIGURE 18 29

SUPPLEMENTARY TABLES 30

SUPPLEMENTARY TABLE 1 30
SUPPLEMENTARY TABLE 2 31
SUPPLEMENTARY TABLE 3 32

SUPPLEMENTARY TABLE 4....33
SUPPLEMENTARY TABLE 5....34
SUPPLEMENTARY TABLE 6....35
SUPPLEMENTARY TABLE 7....36
SUPPLEMENTARY TABLE 8....37
SUPPLEMENTARY TABLE 9....38
SUPPLEMENTARY TABLE 10....39
SUPPLEMENTARY TABLE 11....40
SUPPLEMENTARY TABLE 12....41
SUPPLEMENTARY TABLE 13....42

SUPPLEMENTARY REFERENCES 43

Supplementary Methods

Design Protocol of RNA Constructs

Design of T1-short RNA

To design T1-short RNA, we started with a template construct shown in Figure 1a. For convenience, we define Watson-Crick base pairs G-C and A-U, together with G·U wobbles, as 'strong base pairs', and define G·G, U·U, and G·A as 'weak base pairs'. In the stem region 5′G$X_5X_4X_3X_2X_1$G3′ / 3′A$Y_5Y_4Y_3Y_2Y_1$A5′, X_iY_i represents a strong base pair. The design protocol can be described as follows.

Step 1: All possible sequences for the 5-bp stem were filtered by two criteria: 1) each nucleotide is located in the center of a BP-triplet different from all other nucleotides; 2) upon a single-nucleotide shift in register of the stem, the newly formed base pairs X_2Y_1, X_3Y_2, X_4Y_3, X_5Y_4, and GY_5 should be all strong base pairs or four strong base pairs along with one weak base pair. The first criterion is used to minimize the resonance overlap in the NMR imino 2D spectrum of the RNA, while the second one is to enure the stability of the RNA. A total of 788 hairpins were generated in this step.

Step 2: Each filtered RNA sequence (5′GG$X_5X_4X_3X_2X_1$GCAA$Y_1Y_2Y_3Y_4Y_5$A3′) was subjected to the secondary structure prediction using a standalone version of *MC-Fold*[1] program to obtain the best 100 sub-optimal solutions.

Step 3: A series of filters were applied to the output of *MC-Fold*: 1) GS is the most energetically favored structure; 2) the predefined ES is the second most energetically favored structure; 3) the free energy difference between GS and the predefined ES $\Delta\Delta G \leq 3$ kcal mol^{-1}; 4) there were no more than three consecutive identical bases in the sequence. The last filter was used not only to reduce the overlap of NMR spectral resonances via increasing the variation of a sequence, but also to prevent the design with a relatively long segment containing repeatedly the same base pair that could promote the base pair slipping in either direction.

Step 4: The qualified RNA sequences from steps 1-3 (33 in total) were compiled into a candidate list, which was sorted in ascending order of $\Delta\Delta G$. These sequences were chosen for experimental verification in that order. For T1-short RNA, the first candidate works as expected, making it unnecessary to test other candidates.

Design of T1-derived RNAs

To design T1 RNA, we first kept the T1-short RNA (delete dangling guanine in 5′-end) in the upper hairpin region. The lower stem was fixed to the alternative G-C and C-G base pairs. The variable 3 × 3 internal loop is composed of three mismatches that can be anyone from AA, AC, CA, UC, CU, and CC, leading to a total of 216 (= 6 × 6 × 6) combinations. The next steps are the same as those of T1-short RNA. The secondary structures of these primary sequences were then predicted by *MC-Fold* using the same parameters. The free energy differences

$\Delta\Delta G$ between GS and the predefined ES were ranked. The sequences on the top of the candidate list were then in vitro transcribed for NMR measurements. In our practice, we tested three constructs from the candidate list in the same batch: two of them failed, but the one with AAA/AAA internal loop worked as expected (Supplementary Fig. 2f). We did not continue testing other constructs in the candidate list, but among them many more working constructs presumably will exist.

To design T1-GAAA and T1-UUCG RNA, the apical loop GCAA in T1 RNA was replaced with GAAA and UUCG, respectively. The residues in other places remain the same.

On the basis of T1-short RNA, we removed one base pair (G2·A17, G3-C16, A4-U15, A5-U14, G6·U13, or G7-C12) from the stem region, resulting in five different sequences. The subsequent screening steps are similar to those of T1-short RNA. These sequences were then fed into *MC-Fold* to predict possible secondary structures. The free energy differences $\Delta\Delta G$ between GS and the expected ES were calculated and ranked. Finally, the AAA/AAA internal loop and lower stem sequence of T1 RNA were appended to the newly generated upper hairpins.

To design T1-add1bp, we inserted a G·U base pair on each side of G2·A17 of T1-short in order to minimize the perturbation on the secondary structure switch, resulting in two sequences. Finally, the AAA/AAA internal loop and the lower stem were appended.

To design T1-add2bp RNA, we exhausted all possibilities when inserting a strong base pair (G-C, C-G, A-U, U-A, G·U, or U·G) to the up stem region of T1-add1bp and obtained 31 sequences. The following screening steps are similar to those of T1-short RNA. The secondary structures of these primary sequences were then predicted by *MC-Fold*. The free energy differences $\Delta\Delta G$ between GS and the expected ES were ranked. The RNA sequence corresponding to the smallest $\Delta\Delta G$ was selected. Finally, the AAA/AAA internal loop and the lower stem were appended.

Design of T2-mirror and T2 RNA

To obtain T2-mirror RNA, we swapped two strands of the upper stem of T1 RNA while keeping the GCAA tetraloop, the AAA/AAA internal loop, and the lower stem unchanged.

The design principle and filtering conditions for T2 RNA are similar to those of T1-short. In the upper stem region of GS, base pairs shown in Figure 4b (red) were all strong base pairs. The specific design steps can be described as follows.

Step 1: All possible sequences for the 5-bp stem were filtered by two criteria: 1) each nucleotide is located in the center of a BP-triplet different from all other nucleotides; 2) upon a single-nucleotide shift in the opposite direction compared to T1-short RNA, the reformed base pairs should be all strong base pairs or four strong base pairs along with one weak base pair. A total of 485 RNA constructs were obtained after this step.

Step 2: Each filtered RNA sequence was subjected to the secondary structure prediction using *MC-Fold* to obtain the best 100 sub-optimal solutions.

Step 3: Two filters were applied to the output of *MC-Fold*: 1) GS is the most energetically favored structure; 2) the predefined ES is the second most energetically favored structure.

Step 4: The qualified RNA sequences from steps 1-3 (257 in total) were compiled into a candidate list, which was sorted in ascending order of $\Delta\Delta G$. These sequences were selected for experimental confirmation in that order. And this is followed by the addition of an AAA/AAA internal loop and the lower stem.

Design of T3 RNA

To design T3 RNA, we first fixed GCAA apical loop, residues G11 and A26, 3 × 3 internal loop, and the lower stem as the same as T1 RNA. In the variable upper stem region $5'X_6GX_5X_4X_3X_2X_1G3'/3'AY_5Y_4Y_3Y_2Y_1A5'$, X_iY_i

represents a strong base pair and X_6 represents any one of A, U, G and C. The design protocol can be described as follows.

Step 1: All possible sequences for the 5-bp stem were filtered by two criteria: 1) each nucleotide is located in the center of a BP-triplet different from all other nucleotides; 2) upon a two-nucleotide shift in register of the stem, the newly formed six base pairs X_6Y_5, GY_4, X_5Y_3, X_4Y_2, X_3Y_1 and X_2A should be at least four strong base pairs and all pairs were extracted only from either strong or weak base pair pool; 3) for T3 RNA, the reshuffling base pairs including G11A26→$G11Y_4$, X_5Y_5→X_5Y_3, X_4Y_4→X_4Y_2, X_3Y_3→X_3Y_1, X_2Y_2→X_2A20, X_1Y_1→X_1A26. The number of residues for which the dynamics can be detected by NMR RD experiments (G-C→G·U, U-A→U·G, G-C→G·G, G-C→G·A, and U-A→U·U) must be ≥3 and at least one of the residues undergone the G-C→G·U or U-A→U·G switch. A total of 411 RNA constructs were obtained after this step.

Step 2: Each filtered RNA sequence (5′$X_6GX_5X_4X_3X_2X_1$GCAA$Y_1Y_2Y_3Y_4Y_5$A3′) was subjected to the secondary structure prediction using *MC-Fold* to obtain the best 100 sub-optimal solutions.

Step 3: Two filters were applied to the output of *MC-Fold*: 1) GS is the most energetically favored structure; 2) the predefined ES is the second most energetically favored structure.

Step 4: The qualified RNA sequences from steps 1-3 (94 in total) were compiled into a candidate list, which was sorted in ascending order of $\Delta\Delta G$. These sequences were chosen for experimental verification in that order. And the internal loop and lower stem sequence of T1 RNA would be further added to the regenerated upper hairpin.

Design of T4 RNA

To design T4 RNA, we first fixed the UUCG apical loop, the upper stem made of G-C and C-G alternative base pairs, the 5′GUG/CC3′ motif that is one of the most frequently occurring 1-nt bulge motifs from CoSSMos database[2], and terminal residues G1 and C29. In the regenerated lower stem region 5′$GX_4X_3X_2X_1$G3′/3′$CY_4Y_3Y_2Y_1$A5′, X_iY_i represents a strong base pair. The design protocol can be described as follows.

Step 1: All possible sequences for the 4-bp stem were filtered by two criteria: 1) each nucleotide is located in the center of a BP-triplet different from all other nucleotides; 2) upon a one-nucleotide shift in register of the lower stem, the newly generated five base pairs GY_1, X_1Y_2, X_2Y_3, X_3Y_4, and X_4C should be at least four strong base pairs and all pairs were extracted only from either strong or weak base pair pool. A total of 27 RNA constructs were obtained after this step.

Step 2: Each filtered RNA sequence (5′$GX_4X_3X_2X_1$GUGCGCGCUUCGGCGCGCC$Y_1Y_2Y_3Y_4$C3′) was subjected to the secondary structure prediction using *MC-Fold* to obtain the best 100 sub-optimal solutions.

Step 3: Two filters were applied to the output of *MC-Fold*: 1) GS is the most energetically favored structure; 2) the desired ES is the second most energetically favored structure.

Step 4: The qualified RNA sequences from steps 1-3 (27 in total) were compiled into a candidate list, which was sorted in ascending order of $\Delta\Delta G$. These sequences were chosen for the later NMR experimental verification in that order.

In addition to choosing candidate sequences with the lowest $\Delta\Delta G$s, we also took the following factors into consideration when deciding which constructs will be sent for NMR measurements: 1) the resonances should be dispersed well on 2D imino spectra predicted by imino chemical shift predictor; 2) the priority will be given to RNA constructs with GS-to-ES transition involving base-pair switching between GC and GU, or between UA and UG, in order to ensure pronounced NMR relaxation dispersion signals.

Accelerated Molecular Dynamics (aMD) Simulations

All MD simulations were conducted using *Amber* 18 package with the ff99OL3 force field.[3] To facilitate the conformational switching, the initial conformation was set to the ES structure of each RNA (including T4, T1, and T2). These ES structures were generated using *MC-Fold*/*MC-Sym* pipeline. Each resulting structure was further refined by performing two-stage energy minimization with GPU-accelerated pmemd program in *Amber*.

Specifically, each structure was subjected to the energy minimization for 1000 steps with harmonic restraints applied to heavy atoms (force constant 500 kcal mol^{-1} $Å^{-2}$), followed by 1000 steps of unrestrained minimization. The refined structure with the lowest energy was chosen for the following MD simulations. Specifically, the structure was solvated in a truncated octahedral box (SPC/E water model) whose size was chosen such that the boundary of the box was at least 10 Å away from any of the RNA atoms. The hydrated system was neutralized by adding Na^+ ions and subjected to the energy minimization as described above, then heated from 0 K to the desired temperature followed by 1 ns of equilibrium. The production-stage cMD (conventional Molecular Dynamics) simulations were conducted at 283 K for 400 ns using the NPT ensemble. During the simulations, all bonds involving hydrogen atoms were constrained using the SHAKE algorithm. The nonbonded cutoff was set to 9 Å, and the integration step was 4 fs. The atomic coordinates were stored every 1 ps. The aMD simulations were set up following the published protocol.[4] We used parameter iamd=2 to perform the 'dihedral boost' only. The alpha value for 'dihedral boost' was set to 0.1. The average dihedral energy was computed from the 400th ns of cMD. The trajectories were viewed in *PyMOL* (DeLano Scientific LLC) and *VMD*[5], and were analyzed by *DSSR*[6].

Kinetic Simulations

The kinetic process for T1 RNA reshuffling can be described as a six-state pathway, as shown in Figure 7a and Movie S4. The kinetic differential equation is shown below.

$$\frac{d}{dt}\begin{pmatrix} p_{\mathrm{G}} \\ p_{\mathrm{I_1}} \\ p_{\mathrm{I_2}} \\ p_{\mathrm{I_3}} \\ p_{\mathrm{I_4}} \\ p_{\mathrm{E}} \end{pmatrix} = \begin{pmatrix} -k_{\mathrm{GI_1}} & k_{\mathrm{I_1G}} & 0 & 0 & 0 & 0 \\ k_{\mathrm{GI_1}} & -k_{\mathrm{I_1G}} - k_{\mathrm{I_1I_2}} & k_{\mathrm{I_2I_1}} & 0 & 0 & 0 \\ 0 & k_{\mathrm{I_1I_2}} & -k_{\mathrm{I_2I_1}} - k_{\mathrm{I_2I_3}} & k_{\mathrm{I_3I_2}} & 0 & 0 \\ 0 & 0 & k_{\mathrm{I_2I_3}} & -k_{\mathrm{I_3I_2}} - k_{\mathrm{I_3I_4}} & k_{\mathrm{I_4I_3}} & 0 \\ 0 & 0 & 0 & k_{\mathrm{I_3I_4}} & -k_{\mathrm{I_4I_3}} - k_{\mathrm{I_4E}} & k_{\mathrm{EI_4}} \\ 0 & 0 & 0 & 0 & k_{\mathrm{I_4E}} & -k_{\mathrm{EI_4}} \end{pmatrix} \begin{pmatrix} p_{\mathrm{G}} \\ p_{\mathrm{I_1}} \\ p_{\mathrm{I_2}} \\ p_{\mathrm{I_3}} \\ p_{\mathrm{I_4}} \\ p_{\mathrm{E}} \end{pmatrix} \quad (1)$$

where p_{G} is the population of the T1 RNA GS, $p_{\mathrm{I_1}}, p_{\mathrm{I_2}}, p_{\mathrm{I_3}}, p_{\mathrm{I_4}}$ are populations of I_1-I_4 intermediates, p_{E} is the ES population of T1 RNA, and k_{ij} is the exchange rate constant between states i and j. k_{ij} is calculated based on the following Eyring equation:

$$k_{ij} = \frac{\kappa k_B T}{h} e^{-\frac{\Delta G_{ij}^{\dagger}}{RT}} \quad (2)$$

where κ is the transmission coefficient (assumed to be 1), k_B is the Boltzmann's constant, T is the absolute temperature in Kelvin, h is Planck's constant, $\Delta G_{ij}^{\dagger}$ is the Gibbs free energy of activation from state i to j , and R is the gas constant. The initial condition at t = 0 is set to be $p_{\mathrm{G}} = 1$ and $p_{\mathrm{I_1}} = p_{\mathrm{I_2}} = p_{\mathrm{I_3}} = p_{\mathrm{I_4}} = p_{\mathrm{E}} = 0$.

To obtain the apparent forward (k_{EG}) and reverse (k_{EG}) rate constants, the kinetic reaction is simplified to contain only two states (GS and ES). k_{GE} and k_{EG} are derived from the simultaneous equations $\bar{k}_{\mathrm{ex}} = k_{\mathrm{GE}} + k_{\mathrm{EG}}$ and $p'_{\mathrm{E}}/p'_{\mathrm{G}} = k_{\mathrm{GE}}/k_{\mathrm{EG}}$, where $\bar{k}_{\mathrm{ex}}$ is the average value of exchange rates from the exponential fitting of the $p_{\mathrm{G}}(t)$ and $p_{\mathrm{E}}(t)$ curves, p'_{G} is the population of the T1 GS from the exponential fitting of the $p_{\mathrm{G}}(t)$ curve, and p'_{E} is the population of the T1 ES from the exponential fitting of the $p_{\mathrm{E}}(t)$ curve. Hence the apparent activation energy of T1 RNA can be calculated by equation (2), i.e., $\Delta G_{ij}^{\dagger} = -RT\ln((k_{ij}h)/(\kappa k_B T))$. Finally, the free energy barrier is fine-tuned until the following condition is met: the apparent activation energy $\Delta G_{\mathrm{GE}}^{\dagger}$ is equal to the experimental value of 14.74 kcal mol^{-1} (Figure 5). Analyses and simulations were performed using the in-house python scripts.

Supplementary Figures

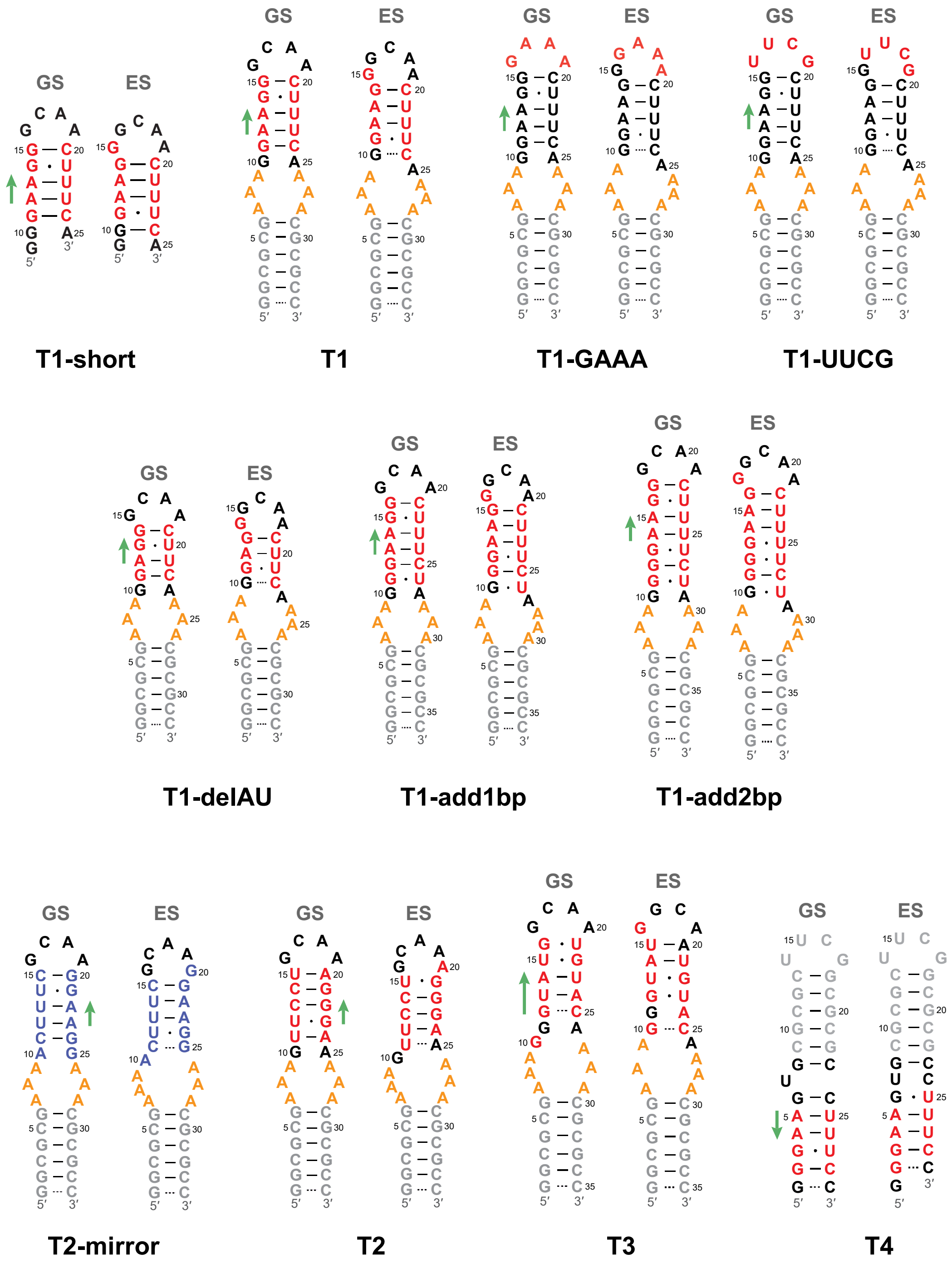

Supplementary Figure 1. The ground state (GS) and excited state (ES) secondary structures of all designed RNAs.

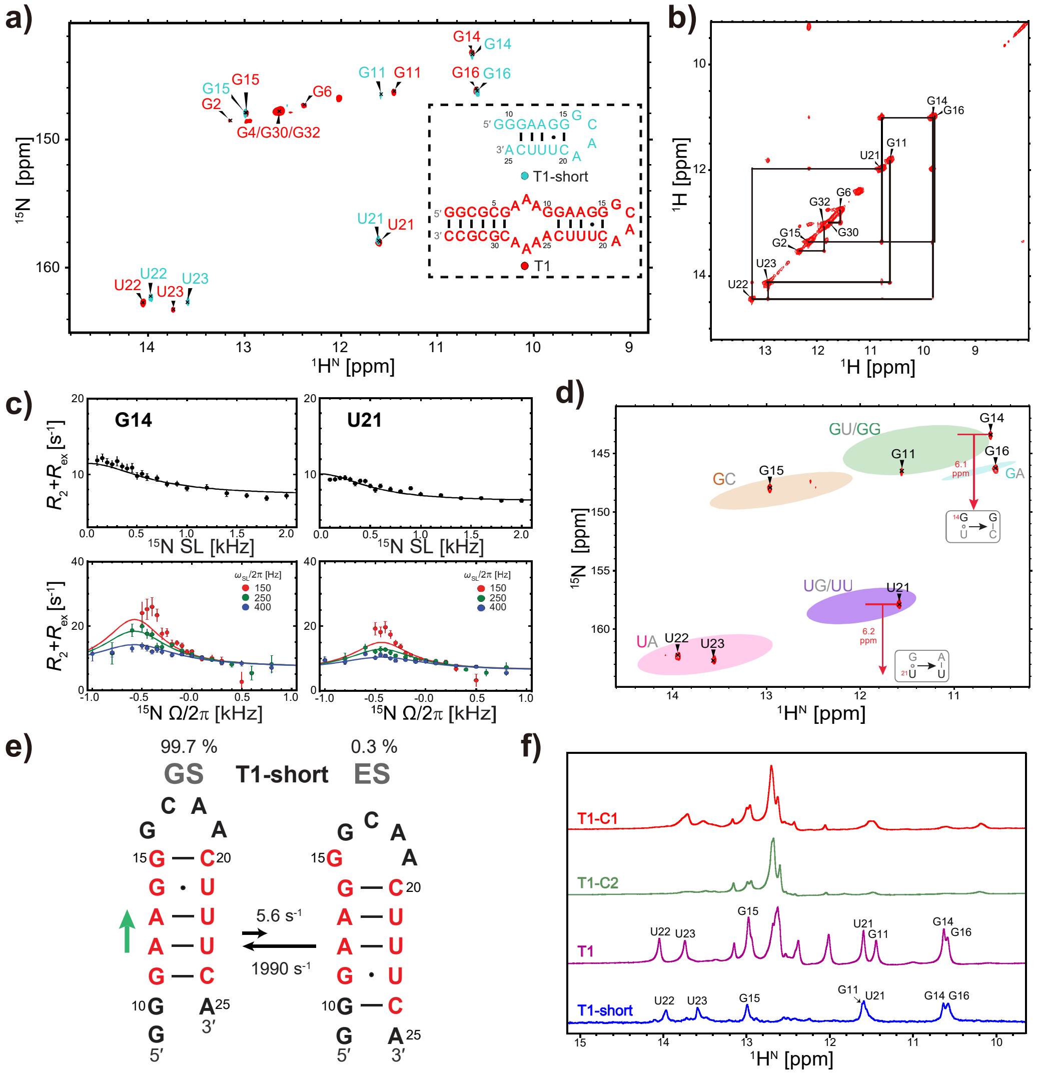

Supplementary Figure 2. **(a)** Overlaid 2D imino SOFAST HMQC spectra of unlabeled T1-short (cyan) and unlabeled T1 (red). Secondary structures are shown as the inset. **(b)** ^{15}N-edited NOESY spectrum showing NOE connectivities of T1 RNA (mixing time: 180 ms). **(c)** Representative on- and off-resonance ^{15}N RD profiles at 10 °C showing the dependence of R_2+R_{ex} on spin-lock power ($\boldsymbol{\omega}_{eff}/2\boldsymbol{\pi}$) and offset ($\Omega/2\pi$) respectively, where Ω is the difference between the carrier frequency of spin-lock pulse and the frequency of the observed resonance. The individual two-state fitting of G14 and U21 gives rise to similar ES population p_B (0.39 % for G6 and 0.23 % for U13) and exchange rate k_{ex} (1596 s^{-1} for G14 and 1554 s^{-1} for U21), suggesting a concerted exchange process. The global fitting reveals an ES with a population of 0.28 ± 0.02 % and a lifetime of 0.5 ± 0.1 ms. The error bars in ^{15}N RD profiles represent standard deviations (SD) estimated using Monte Carlo simulation with 50 iterations. **(d)** 2D ^{1}H-^{15}N HMQC spectrum of T1-short. The residues showing pronounced RD are highlighted by red arrows starting at ^{15}N chemical shift of GS and ending at ^{15}N chemical shift of ES, and are labeled with the associated $\Delta\omega$ from individual fitting. Also shown is the ellipsoid distribution of ^{15}N and ^{1}H^{N} chemical shifts of RNA imino groups in different base pair contexts. The chemical shift data are extracted from the BMRB database.[7] The ellipses are centered on the average chemical shift and have the size that corresponds to twice the standard deviation (SD) in the distribution. **(e)** GS and ES structures of T1-short, along with their populations and interconverting rate constants. The residues in the variable stem are shown in red. The green arrow on the left indicates the direction of sliding. **(f)** Imino region of the 1D proton spectra of the T1-C1, T1-C2, T1, and T1-short RNAs. T1-C1 and T1-C2 RNAs contain the first two loops in Fig. 1d, respectively. Assignments are annotated for the imino proton resonances of T1 upper hairpin region and T1-short RNA. All NMR experiments were conducted at 10 °C in 10mM sodium phosphate and 0.01 mM EDTA, pH 6.4.

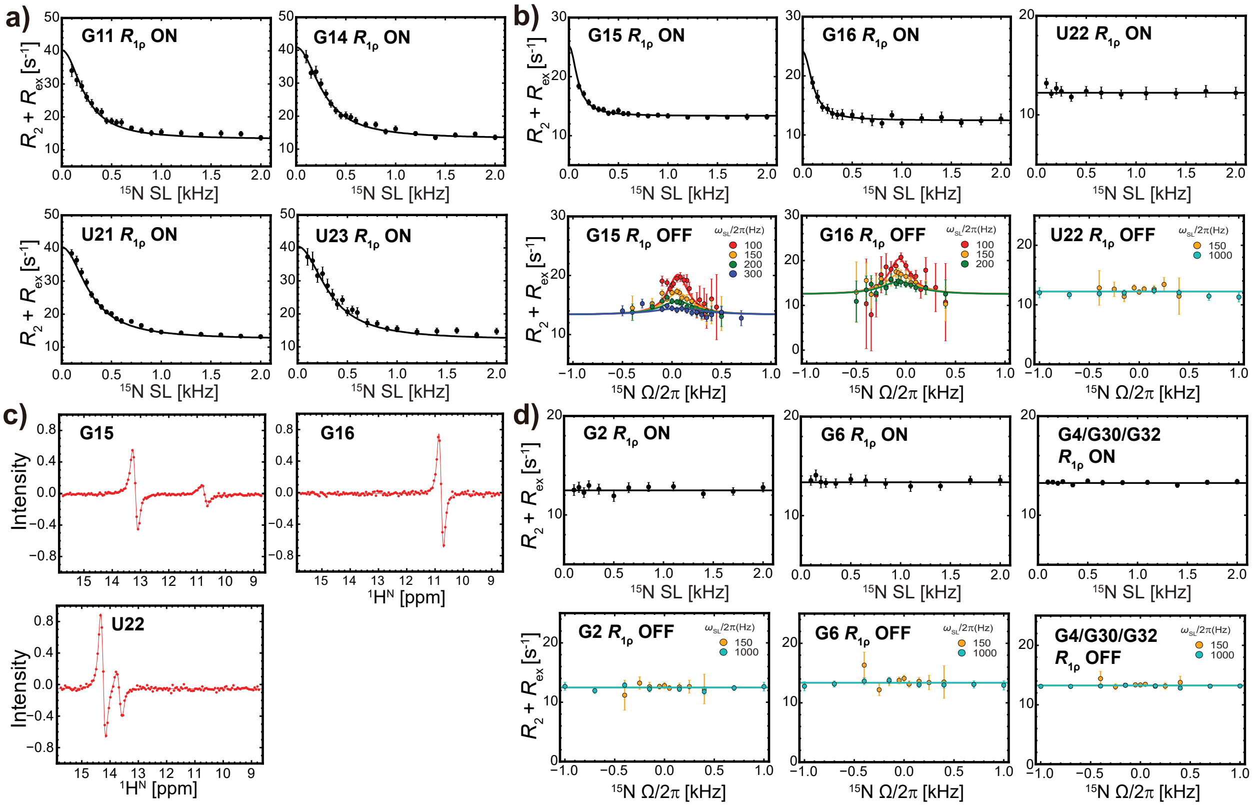

Supplementary Figure 3. ^{15}N $R_{1\rho}$ and ^{1}H^{N} CEST profiles of T1 RNA. **(a)** On-resonance ^{15}N $R_{1\rho}$ profiles of residues G11, G14, U21, and U23. **(b)** On- and off-resonance ^{15}N $R_{1\rho}$ profiles of residues G15, G16, and U22. **(c)** ^{1}H^{N} CEST profiles of residues G15, G16, and U22. **(d)** ^{15}N $R_{1\rho}$ profiles of residues G2, G6, and G4/G30/G32 located in the lower stem of T1 RNA. The error bars in ^{15}N RD profiles represent standard deviations (SD) estimated using Monte Carlo simulation with 50 iterations.

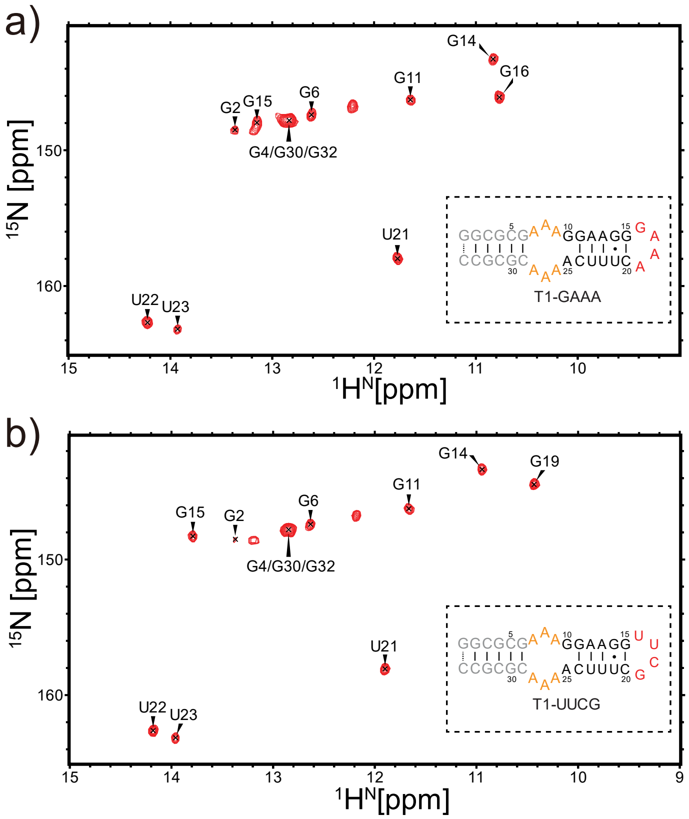

Supplementary Figure 4. 2D imino SOFAST HMQC spectra of T1-GAAA **(a)** and T1-UUCG **(b)** measured at 10 °C. Secondary structures of T1-GAAA and T1-UUCG are shown as the insets.

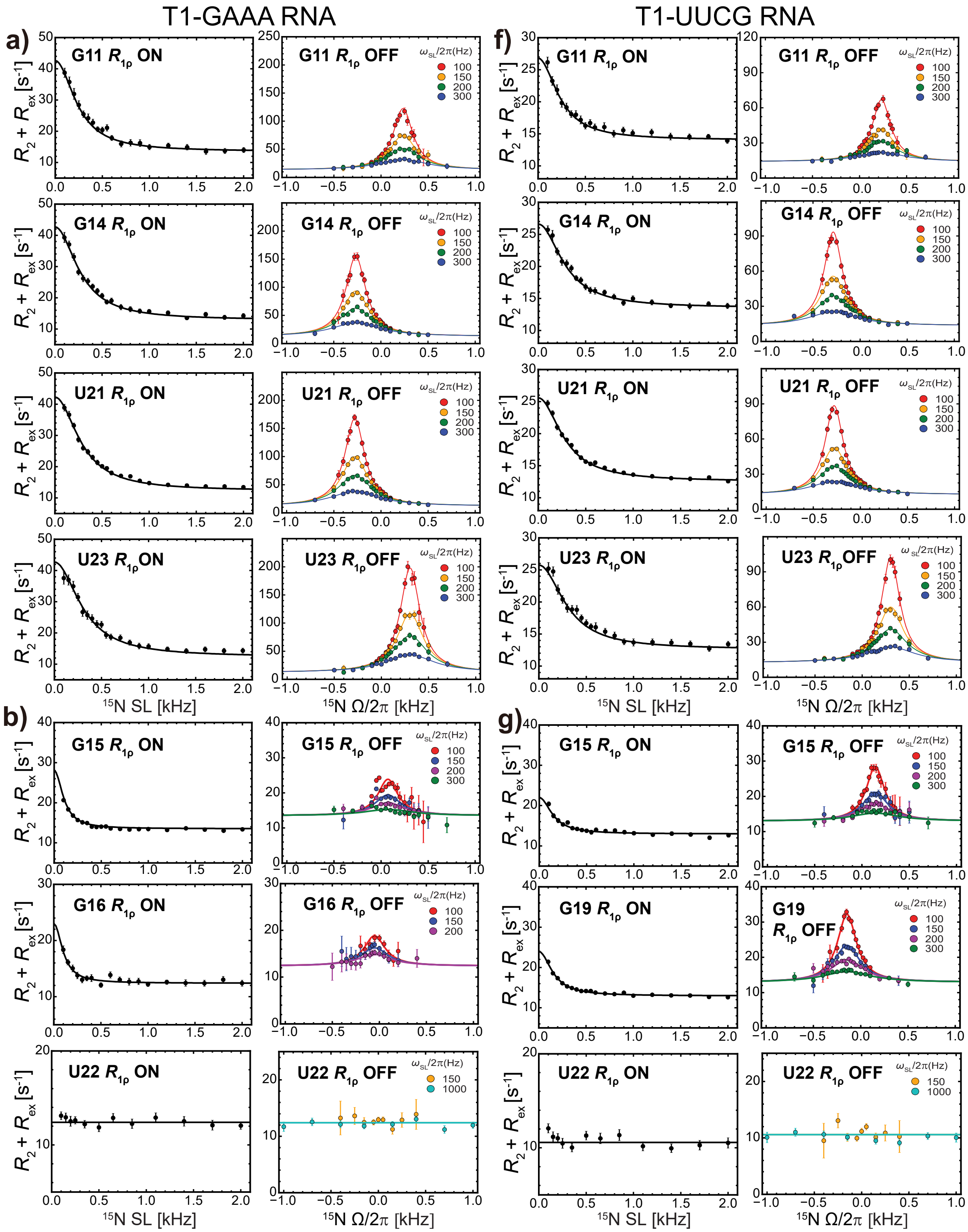
T1-GAAA RNA
T1-UUCG RNA
a)
f)
b)
g)
G11 $R_{1\rho}$ ON
G11 $R_{1\rho}$ OFF
G14 $R_{1\rho}$ ON
G14 $R_{1\rho}$ OFF
U21 $R_{1\rho}$ ON
U21 $R_{1\rho}$ OFF
U23 $R_{1\rho}$ ON
U23 $R_{1\rho}$ OFF
G15 $R_{1\rho}$ ON
G15 $R_{1\rho}$ OFF
G16 $R_{1\rho}$ ON
G16 $R_{1\rho}$ OFF
G19 $R_{1\rho}$ ON
G19 $R_{1\rho}$ OFF
U22 $R_{1\rho}$ ON
U22 $R_{1\rho}$ OFF
$\omega_{SL}/2\pi$(Hz)
100
150
200
300
1000
$R_2 + R_{ex}$ [s^{-1}]
^{15}N SL [kHz]
^{15}N $\Omega/2\pi$ [kHz]

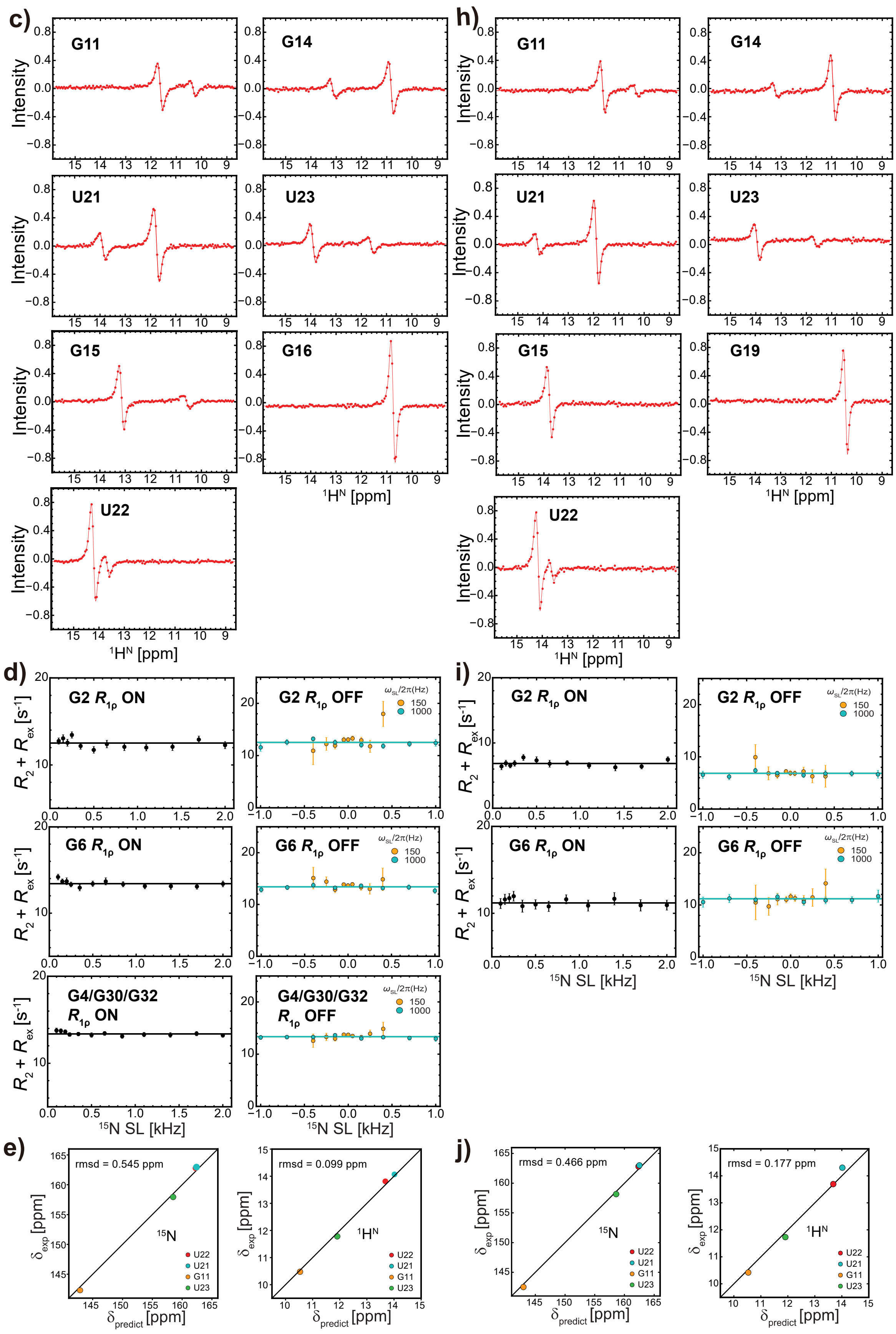

c)
G11
G14
U21
U23
G15
G16
U22
Intensity
$^1H^N$ [ppm]
h)
G11
G14
U21
U23
G15
G19
U22
Intensity
$^1H^N$ [ppm]
d)
G2 $R_{1\rho}$ ON
G2 $R_{1\rho}$ OFF
G6 $R_{1\rho}$ ON
G6 $R_{1\rho}$ OFF
G4/G30/G32 $R_{1\rho}$ ON
G4/G30/G32 $R_{1\rho}$ OFF
$\omega_{SL}/2\pi$(Hz)
150
1000
$R_2 + R_{ex}$ [s^{-1}]
^{15}N SL [kHz]
i)
G2 $R_{1\rho}$ ON
G2 $R_{1\rho}$ OFF
G6 $R_{1\rho}$ ON
G6 $R_{1\rho}$ OFF
$\omega_{SL}/2\pi$(Hz)
150
1000
$R_2 + R_{ex}$ [s^{-1}]
^{15}N SL [kHz]
e)
rmsd = 0.545 ppm
^{15}N
rmsd = 0.099 ppm
$^1H^N$
U22
U21
G11
U23
δ_{exp} [ppm]
$\delta_{predict}$ [ppm]
j)
rmsd = 0.466 ppm
^{15}N
rmsd = 0.177 ppm
$^1H^N$
U22
U21
G11
U23
δ_{exp} [ppm]
$\delta_{predict}$ [ppm]

Supplementary Figure 5. **(a,f)** On- and off-resonance ^{15}N $R_{1\rho}$ profiles of residues showing significant RD signals in T1-GAAA (a) and T1-UUCG (f). **(b,g)** On- and off-resonance ^{15}N $R_{1\rho}$ profiles of other residues in the upper hairpin region of T1-GAAA (b) and T1-UUCG (g). **(c,h)** $^{1}H^{N}$ CEST profiles of residues in the upper hairpin region of T1-GAAA (c) and T1-UUCG (h). **(d,i)** On- and off-resonance ^{15}N $R_{1\rho}$ profiles of residues in the lower stem region of T1-GAAA (d) and T1-UUCG (i). **(e,j)** Correlations between the predicted and the experimental ^{15}N (left) and $^{1}H^{N}$ (right) chemical shifts of ES for T1-GAAA (e) and T1-UUCG (j). The error bars in ^{15}N RD profiles represent standard deviations (SD) estimated using Monte Carlo simulation with 50 iterations.

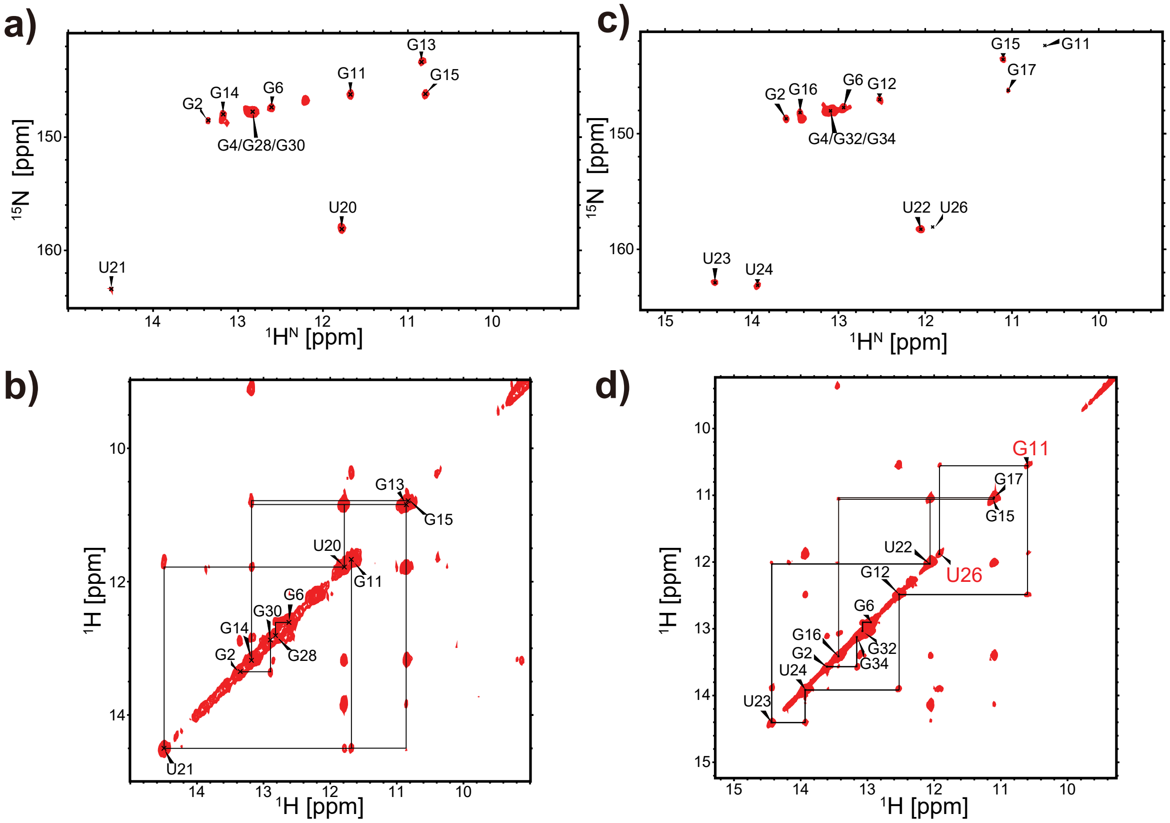

Supplementary Figure 6. (a,c) 2D imino SOFAST HMQC spectra of T1-delAU (a) and T1-add1bp (c) at 10 °C. **(b,d)** ^{15}N-edited NOESY spectra showing NOE connectivities for T1-delAU (b) and T1-add1bp (d) at 10 °C. For T1-add1bp RNA, the labels of inserted residues, G11 and U26, are colored in red. The NOESY mixing time was set to 180 ms.

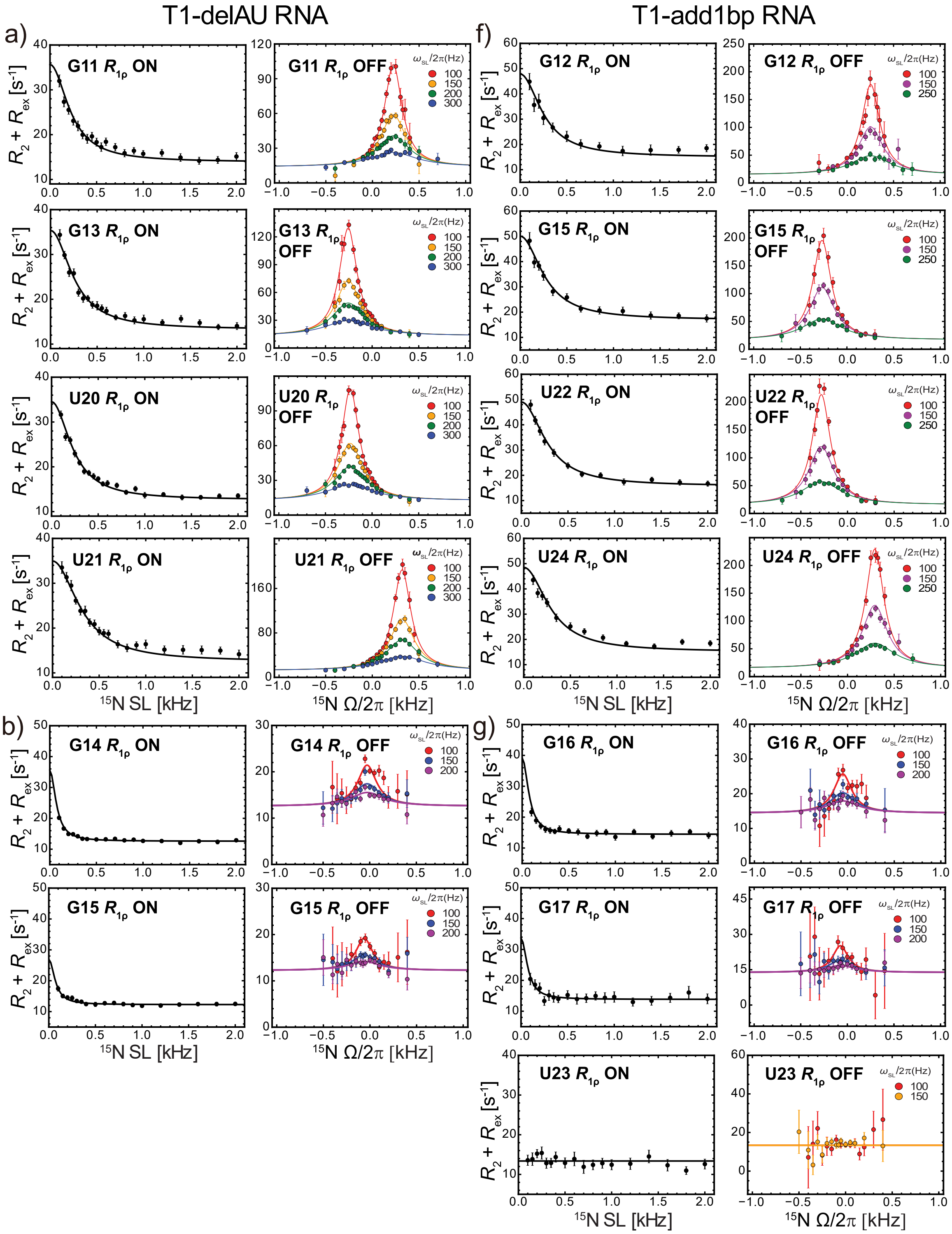

T1-delAU RNA
T1-add1bp RNA
a)
f)
b)
g)
G11 $R_{1\rho}$ ON
G11 $R_{1\rho}$ OFF
G13 $R_{1\rho}$ ON
G13 $R_{1\rho}$ OFF
U20 $R_{1\rho}$ ON
U20 $R_{1\rho}$ OFF
U21 $R_{1\rho}$ ON
U21 $R_{1\rho}$ OFF
G12 $R_{1\rho}$ ON
G12 $R_{1\rho}$ OFF
G15 $R_{1\rho}$ ON
G15 $R_{1\rho}$ OFF
U22 $R_{1\rho}$ ON
U22 $R_{1\rho}$ OFF
U24 $R_{1\rho}$ ON
U24 $R_{1\rho}$ OFF
G14 $R_{1\rho}$ ON
G14 $R_{1\rho}$ OFF
G16 $R_{1\rho}$ ON
G16 $R_{1\rho}$ OFF
G17 $R_{1\rho}$ ON
G17 $R_{1\rho}$ OFF
U23 $R_{1\rho}$ ON
U23 $R_{1\rho}$ OFF
$\omega_{SL}/2\pi$(Hz)
100
150
200
250
300
$R_2 + R_{ex}$ [s^{-1}]
^{15}N SL [kHz]
^{15}N $\Omega/2\pi$ [kHz]

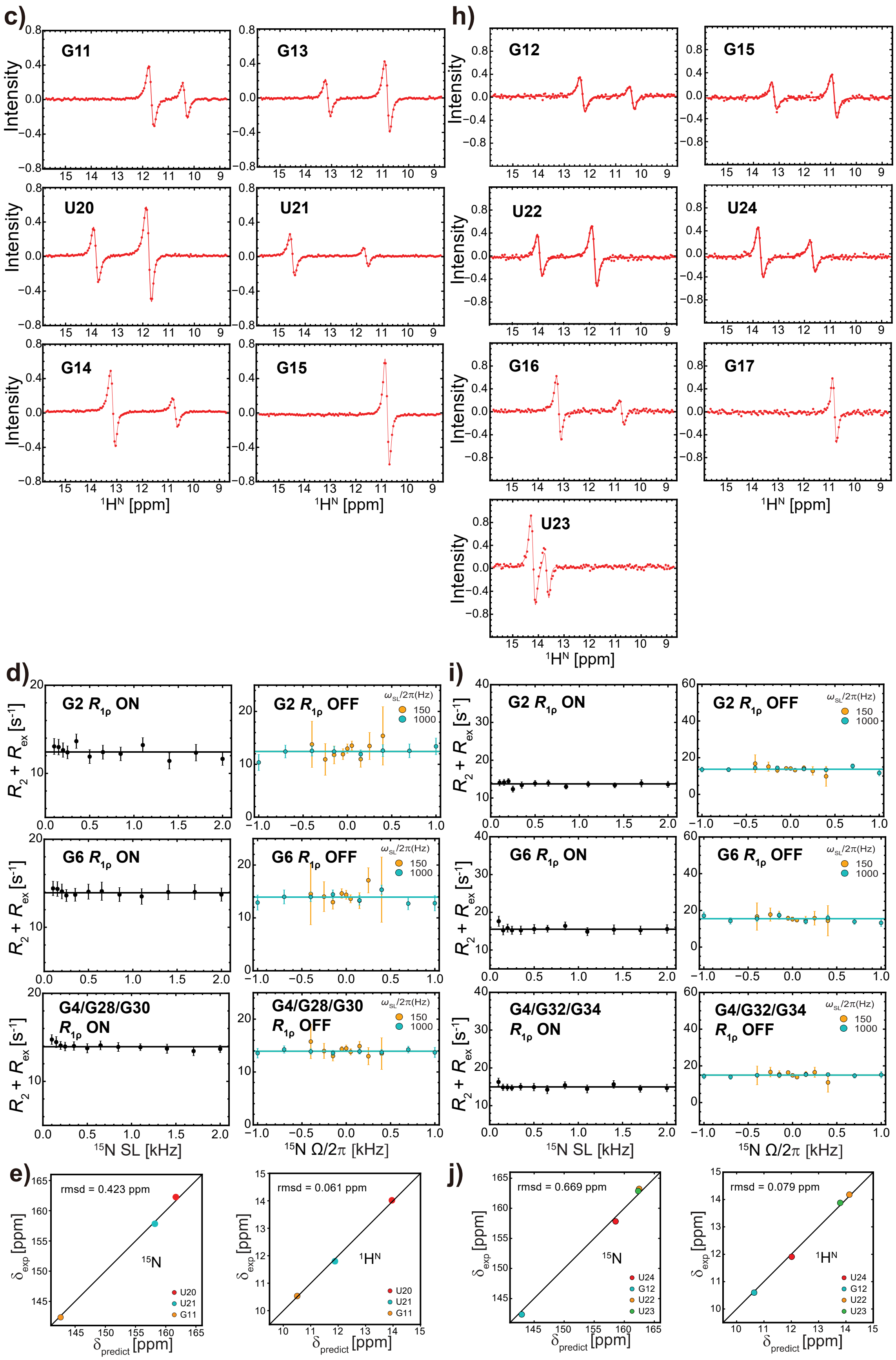
c)
G11
G13
U20
U21
G14
G15
Intensity
^{1}H^N [ppm]
h)
G12
G15
U22
U24
G16
G17
U23
Intensity
^{1}H^N [ppm]
d)
G2 $R_{1\rho}$ ON
G2 $R_{1\rho}$ OFF
G6 $R_{1\rho}$ ON
G6 $R_{1\rho}$ OFF
G4/G28/G30 $R_{1\rho}$ ON
G4/G28/G30 $R_{1\rho}$ OFF
$\omega_{SL}/2\pi$(Hz)
150
1000
$R_2 + R_{ex}$ [s^{-1}]
^{15}N SL [kHz]
^{15}N $\Omega/2\pi$ [kHz]
i)
G2 $R_{1\rho}$ ON
G2 $R_{1\rho}$ OFF
G6 $R_{1\rho}$ ON
G6 $R_{1\rho}$ OFF
G4/G32/G34 $R_{1\rho}$ ON
G4/G32/G34 $R_{1\rho}$ OFF
$\omega_{SL}/2\pi$(Hz)
150
1000
$R_2 + R_{ex}$ [s^{-1}]
^{15}N SL [kHz]
^{15}N $\Omega/2\pi$ [kHz]
e)
rmsd = 0.423 ppm
^{15}N
U20
U21
G11
rmsd = 0.061 ppm
^{1}H^N
U20
U21
G11
δ_{exp} [ppm]
$\delta_{predict}$ [ppm]
j)
rmsd = 0.669 ppm
^{15}N
U24
G12
U22
U23
rmsd = 0.079 ppm
^{1}H^N
U24
G12
U22
U23
δ_{exp} [ppm]
$\delta_{predict}$ [ppm]

Supplementary Figure 7. (a,f) On- and off-resonance ^{15}N $R_{1\rho}$ profiles of residues showing significant RD signals in T1-delAU (a) and T1-add1bp (f). **(b,g)** On- and off-resonance ^{15}N $R_{1\rho}$ profiles of other residues in the upper hairpin region of T1-delAU (b) and T1-add1bp (g). **(c,h)** $^{1}H^{N}$ CEST profiles for residues in the upper hairpin region of T1-delAU (c) and T1-add1bp (h). **(d,i)** On- and off-resonance ^{15}N $R_{1\rho}$ profiles of residues in the lower stem region of T1-delAU (d) and T1-add1bp (i). **(e,j)** Correlations between the predicted and the experimental ^{15}N (left) and $^{1}H^{N}$ (right) chemical shifts of ES for T1-delAU (e) and T1-add1bp (j). The error bars in ^{15}N RD profiles represent standard deviations (SD) estimated using Monte Carlo simulation with 50 iterations.

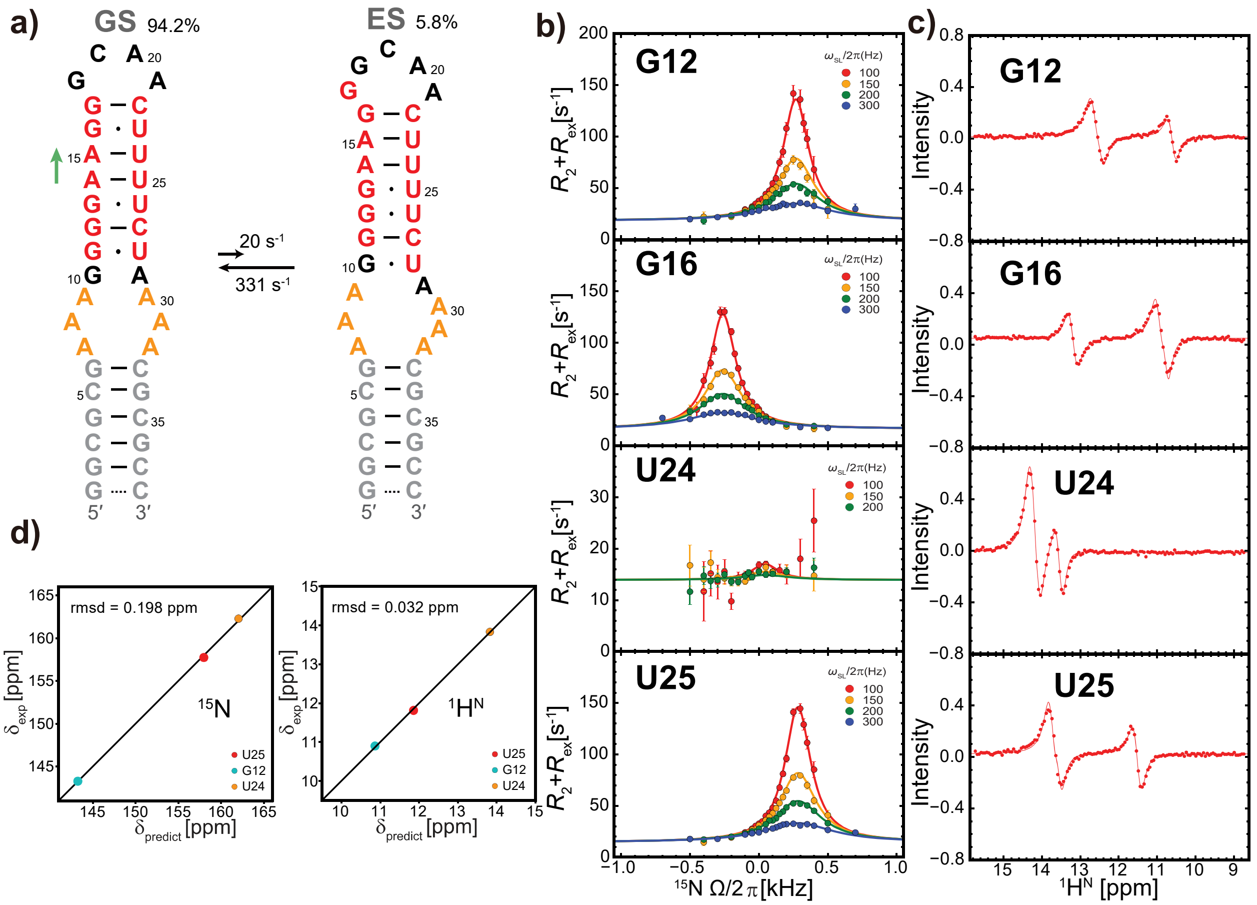

Supplementary Figure 8. The design and verification of T1-add2bp. **(a)** Secondary structure reshuffling of T1-add2bp comprising an elongated upper hairpin (black and red), a 3 × 3 internal-loop (orange), and a stable lower stem composed of alternative G-C and C-G base pairs (gray). The green arrow on the left represents the direction of sliding. **(b)** Off-resonance ^{15}N $R_{1\rho}$ profiles of four representative residues, G12, G16, U24, and U25. The error bars in ^{15}N RD profiles represent standard deviations (SD) estimated using Monte Carlo simulation with 50 iterations. **(c)** ^{1}H^{N} CEST profiles of G12, G16, U24, and U25. **(d)** Correlation between the predicted and the experimental imino ^{15}N (left) and ^{1}H^{N} (right) chemical shifts of ES for T1-add2bp.

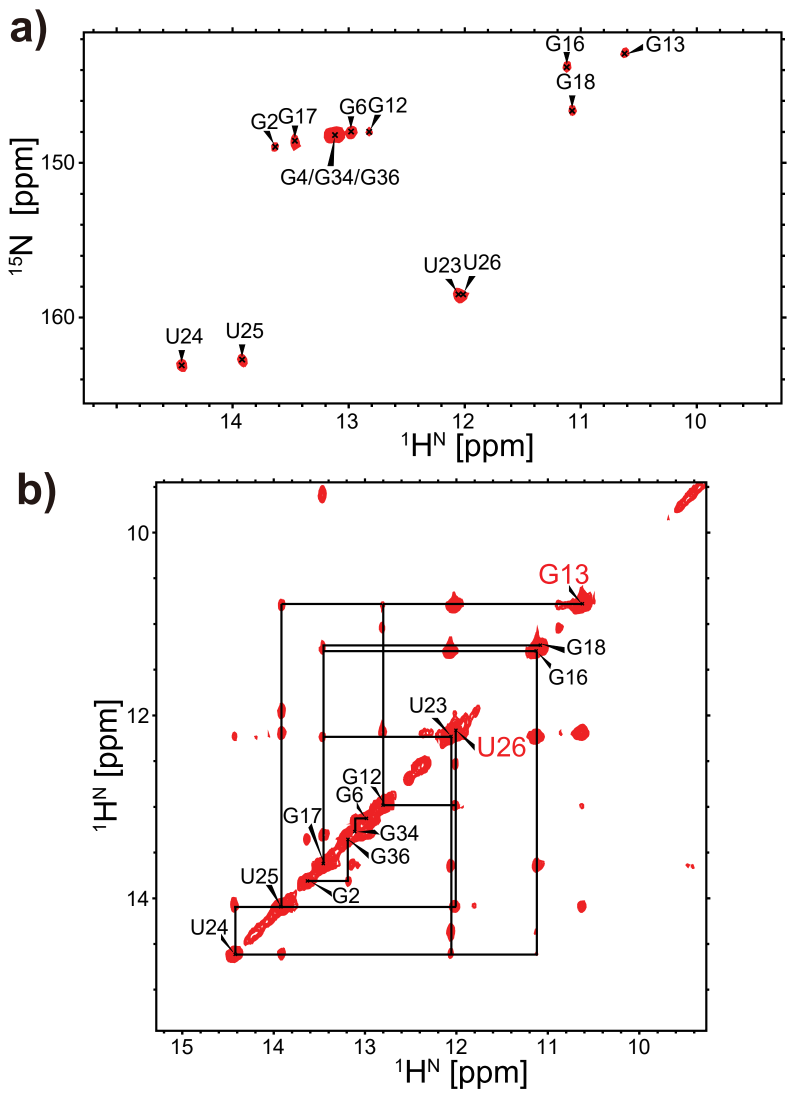

Supplementary Figure 9. (a) 2D imino SOFAST HMQC spectrum of T1-add2bp at 10 °C. **(b)** ^{15}N-edited NOESY spectrum showing NOE connectivities for T1-add2bp at 10 °C. The labels of inserted residues, G13 and U26, are colored in red. The NOESY mixing time was set to 180 ms.

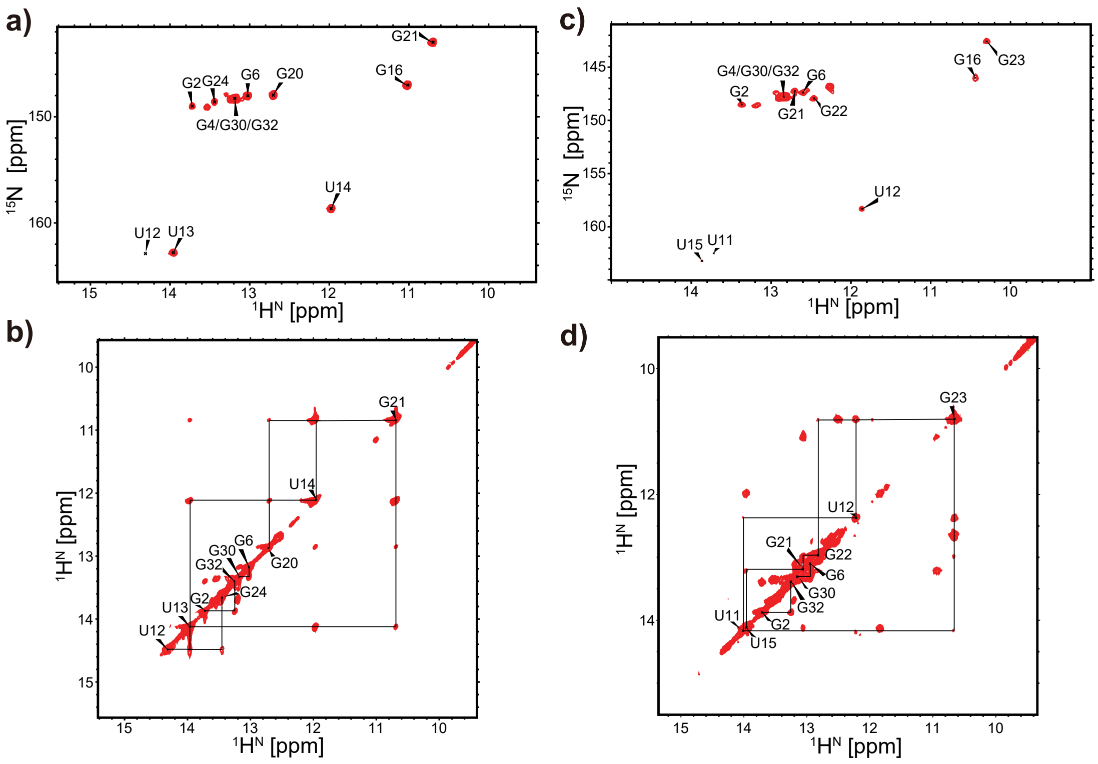

Supplementary Figure 10. (a,c) 2D imino SOFAST HMQC spectra of T2-mirror (a) and T2 (c) at 10 °C. **(b,d)** ^{15}N-edited NOESY spectra showing NOE connectivities for T2-mirror (b) and T2 (d) at 10 °C. The NOESY mixing time was 180 ms.

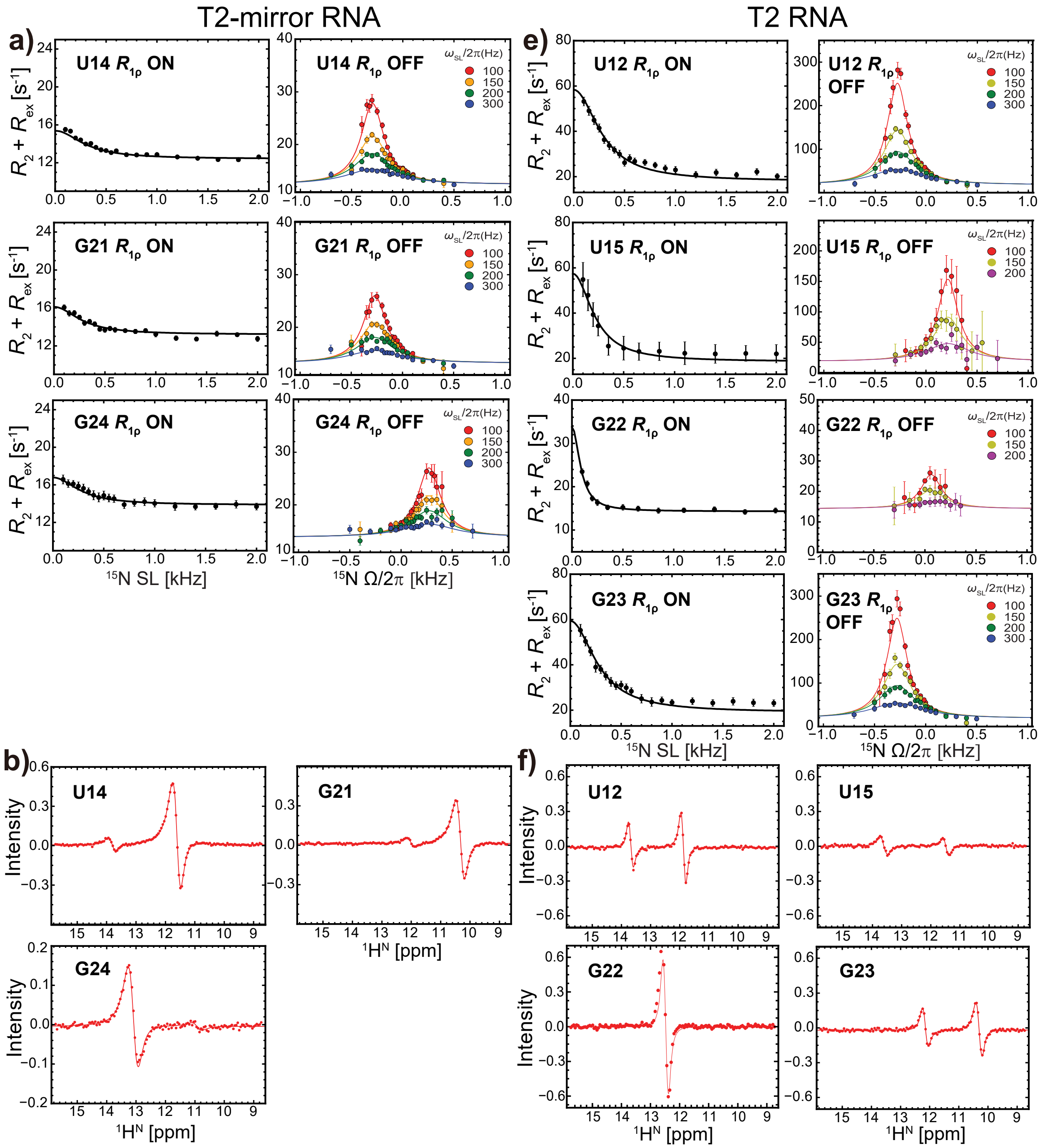

T2-mirror RNA
T2 RNA
a)
U14 $R_{1\rho}$ ON
U14 $R_{1\rho}$ OFF
G21 $R_{1\rho}$ ON
G21 $R_{1\rho}$ OFF
G24 $R_{1\rho}$ ON
G24 $R_{1\rho}$ OFF
$R_2 + R_{ex}$ [s^{-1}]
$\omega_{SL}/2\pi$(Hz)
100
150
200
300
^{15}N SL [kHz]
^{15}N $\Omega/2\pi$ [kHz]
e)
U12 $R_{1\rho}$ ON
U12 $R_{1\rho}$ OFF
U15 $R_{1\rho}$ ON
U15 $R_{1\rho}$ OFF
G22 $R_{1\rho}$ ON
G22 $R_{1\rho}$ OFF
G23 $R_{1\rho}$ ON
G23 $R_{1\rho}$ OFF
b)
U14
G21
G24
Intensity
^{1}H^N [ppm]
f)
U12
U15
G22
G23

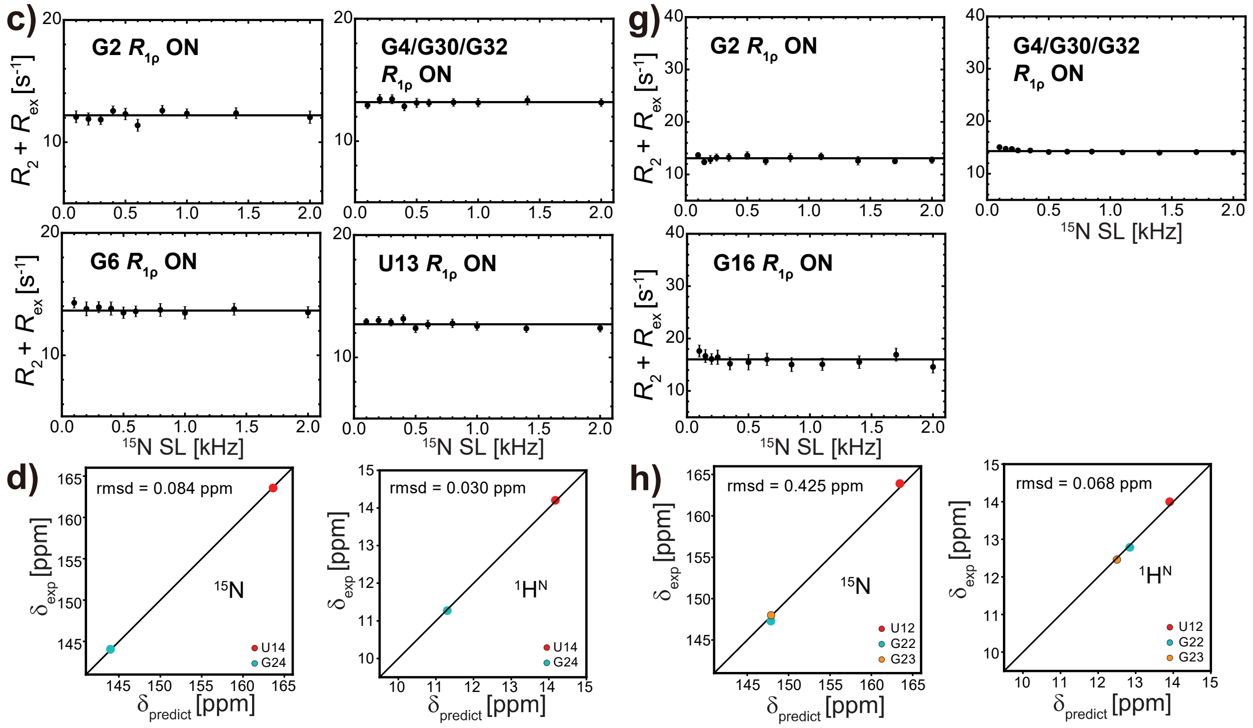

Supplementary Figure 11. (a,e) On- and off-resonance ^{15}N $R_{1\rho}$ profiles of residues showing significant RD signals in T2-mirror (a) and T2 (e). **(b,f)** ^{1}H^{N} CEST profiles for residues in the upper hairpin region of T2-mirror (b) and T2 (f). **(c,g)** On- and off-resonance ^{15}N $R_{1\rho}$ profiles of residues in the lower stem region of T2-mirror (c) and T2 (g). **(d,h)** Correlations between the predicted and the experimental ^{15}N (left) and ^{1}H^{N} (right) chemical shifts of ES for T2-mirror (d) and T2 (h). The error bars in ^{15}N RD profiles represent standard deviations (SD) estimated using Monte Carlo simulation with 50 iterations.

a)

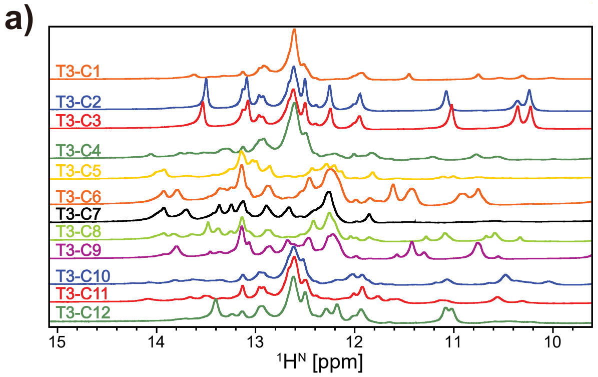

Construct	Sequences	Result
T3-C1	GGCGCGAAAAGCGUGUGCAAACGCGAAAACGCGCC	Incorrect 2nd structure
T3-C2	GGCGCGAAAGGUGCUUGCAAGAGCAAAAACGCGCC	No RD signal
T3-C3	GGCGCGAAAGGUGCUUGAAAGAGCAAAAACGCGCC	No RD signal
T3-C4	GGCGCGAAAGGAUGUGGCAACGUAUAAAACGCGCC	Incorrect 2nd structure
T3-C5	GGCAGAUCUGUAUGGCAAUGUACUCUGCC	Incorrect 2nd structure
T3-C6	GGCAGAUCUGUGUGGCAAUGCACUCUGCC	Incorrect 2nd structure
T3-C7	GGCAGAUCUGUGUGGCAACGUACUCUGCC	Incorrect 2nd structure
T3-C8	GGCAGAUCUGUGUGGCAACAUACUCUGCC	Incorrect 2nd structure
T3-C9	GGCAGAUCUGUGUGGCAACGCACUCUGCC	No RD signal
T3-C10	GGCGCGAAAGGAGGUGGCAACGUCUAAAACGCGCC	Incorrect 2nd structure
T3-C11	GGCGCGAAAGGGGAUGGCAAUGUCCAAAACGCGCC	Incorrect 2nd structure
T3-C12	GGCGCGAAAGGGCAUGGCAAUGUGCAAAACGCGCC	No RD signal

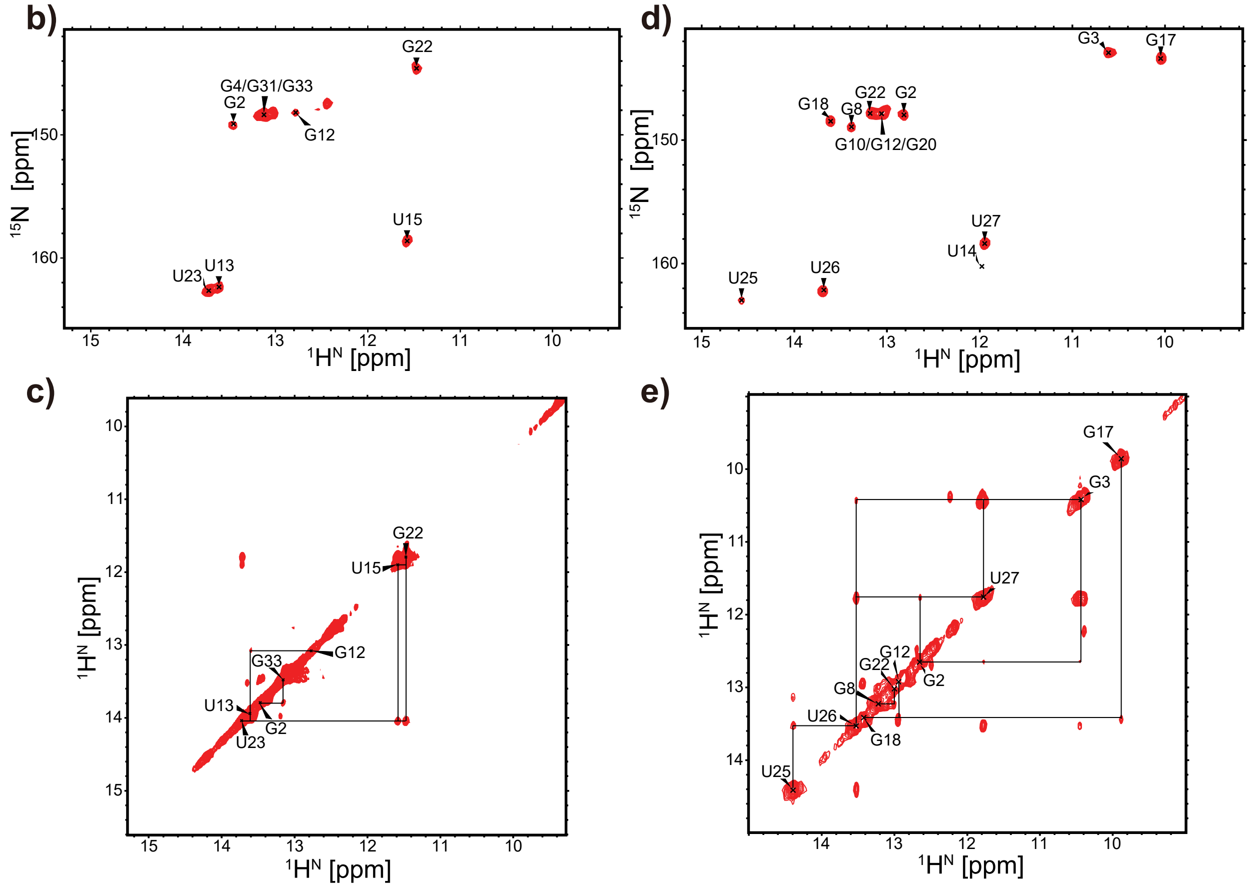

Supplementary Figure 12. (a) Imino region of the 1D proton spectra of 12 failed candidates of T3 RNA (left), and the corresponding sequences and measurement results (right). **(b)** 2D imino SOFAST HMQC spectrum of T3. **(c)** ^{15}N-edited NOESY spectrum showing NOE connectivities for T3. **(d)** 2D imino SOFAST HMQC spectrum of T4. **(e)** ^{15}N-edited NOESY spectrum showing NOE connectivities for T4. The NOESY mixing time was set to 180 ms. All these NMR experiments were conducted at 10 °C.

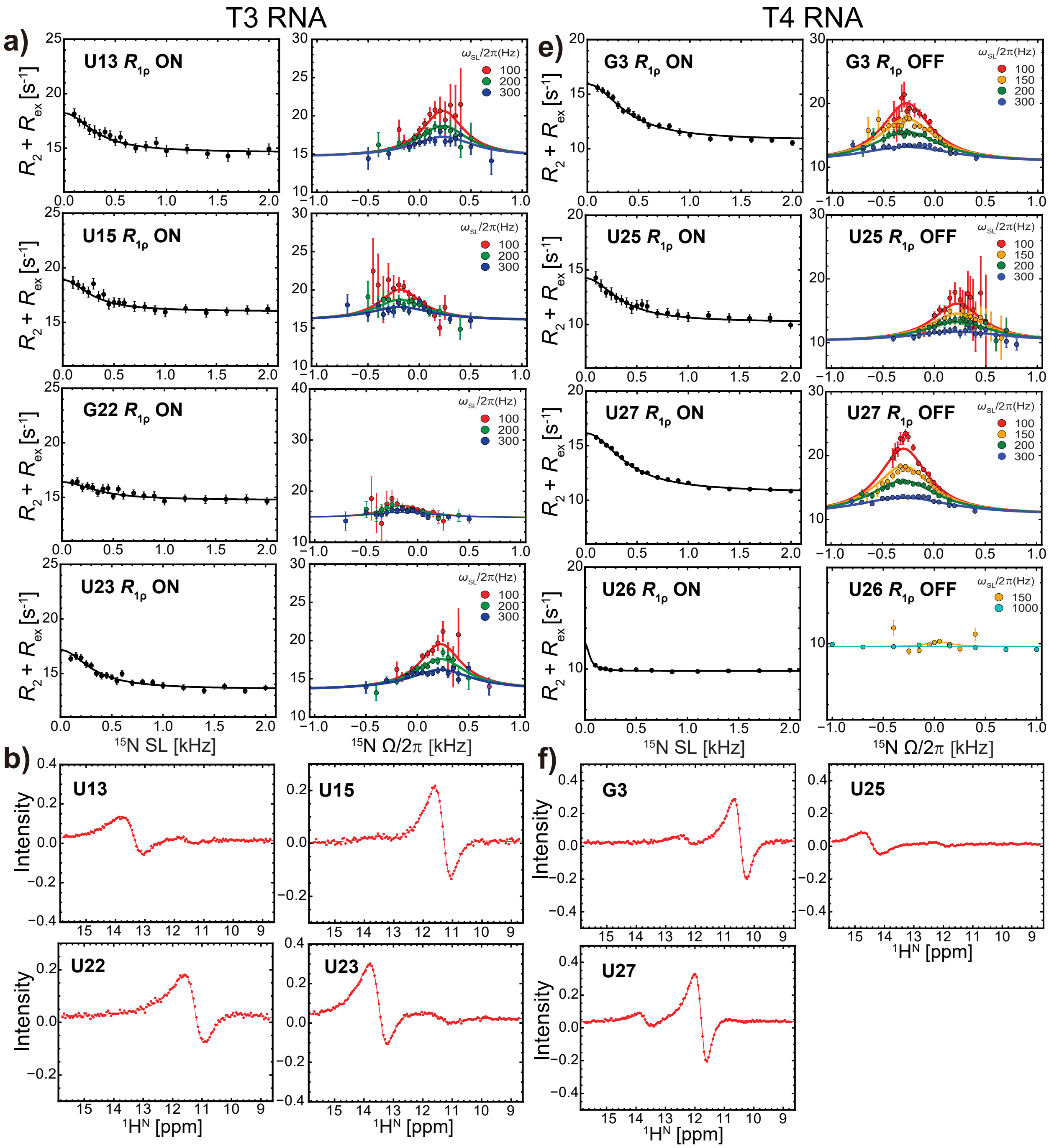
T3 RNA
T4 RNA
a)
e)
b)
f)
U13 $R_{1\rho}$ ON
U15 $R_{1\rho}$ ON
G22 $R_{1\rho}$ ON
U23 $R_{1\rho}$ ON
G3 $R_{1\rho}$ ON
G3 $R_{1\rho}$ OFF
U25 $R_{1\rho}$ ON
U25 $R_{1\rho}$ OFF
U27 $R_{1\rho}$ ON
U27 $R_{1\rho}$ OFF
U26 $R_{1\rho}$ ON
U26 $R_{1\rho}$ OFF
$\omega_{SL}/2\pi$(Hz)
100
150
200
300
1000
$R_2 + R_{ex}$ [s^{-1}]
^{15}N SL [kHz]
^{15}N $\Omega/2\pi$ [kHz]
U13
U15
U22
U23
G3
U25
U27
Intensity
^{1}H^N [ppm]

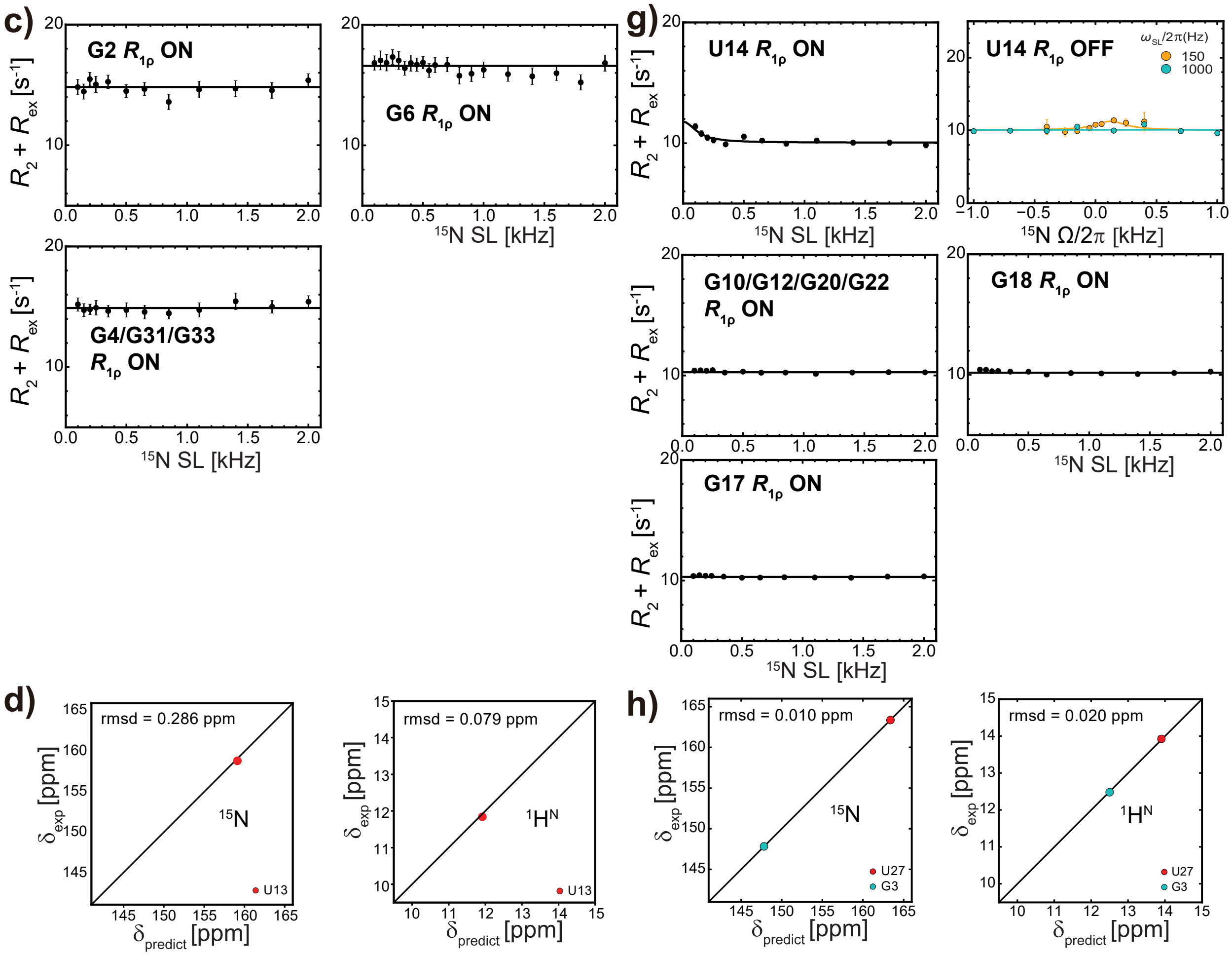

Supplementary Figure 13. **(a,e)** On- and off-resonance ^{15}N $R_{1\rho}$ profiles of residues showing significant RD signals in T3 (a) and T4 (e). The weak RD signal for G22 in T3 RNA is due to the small change in chemical shifts between GS and ES, corresponding to the BP-triplet change from 5′-UG-**G**U-UA-3′ to 5′-UA-**G**U-UG-3′. The marginal RD signal for U26 in T4 RNA is due to the small chemical shift change for the transition from U26^{GS}-A4 to U26^{ES}-A5. **(b,f)** ^{1}H^{N} CEST profiles for residues in T3 (b) and T4 (f). **(c,g)** On- and off-resonance ^{15}N $R_{1\rho}$ profiles of residues in the lower stem region of T3 (c) and in the upper hairpin region of T4 (g). **(d,h)** Correlations between the predicted and the experimental ^{15}N (left) and ^{1}H^{N} (right) chemical shifts of ES for T3 (d) and T4 (h). The error bars in ^{15}N RD profiles represent standard deviations (SD) estimated using Monte Carlo simulation with 50 iterations.

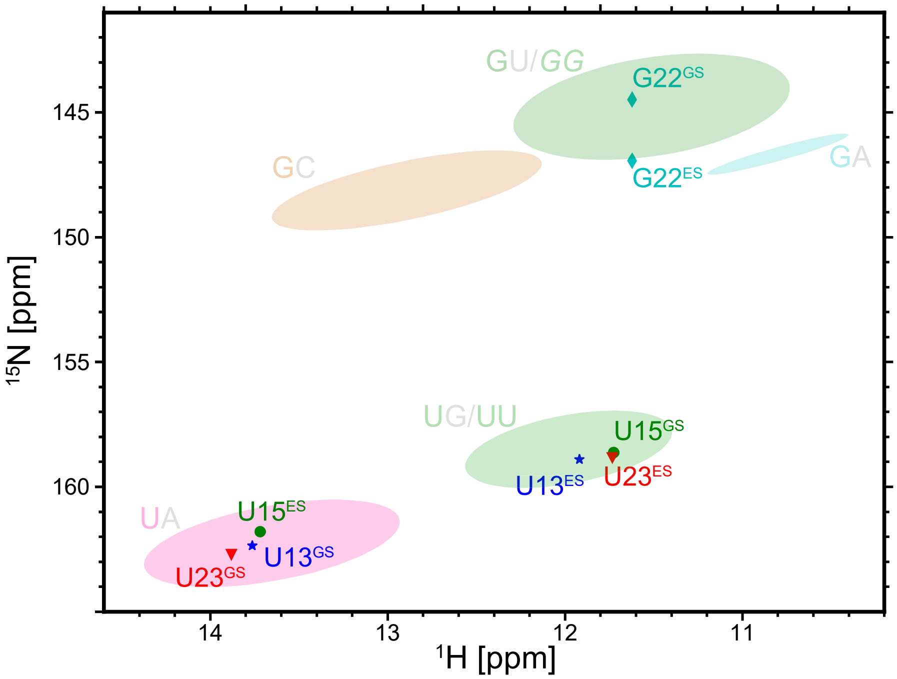

Supplementary Figure 14. GS and ES chemical shifts of residues U13, U15, U23, and G22 in the upper stem of T3 RNA showing transitions between U-A and U·G base pairs, including U13^{GS}-A → U13^{ES}·G, U15^{GS}·G → U15^{ES}-A, U23^{GS}-A → U23^{ES}·G. An exception is G22 that shows a transition of G22^{GS}·U → G22^{ES}·U. Also shown is the ellipsoid distribution of RNA imino resonances for different base pairs, as derived from the BMRB database. The ellipses are centered on the average chemical shift and have a size that corresponds to twice the standard deviation (SD) of the cluster.

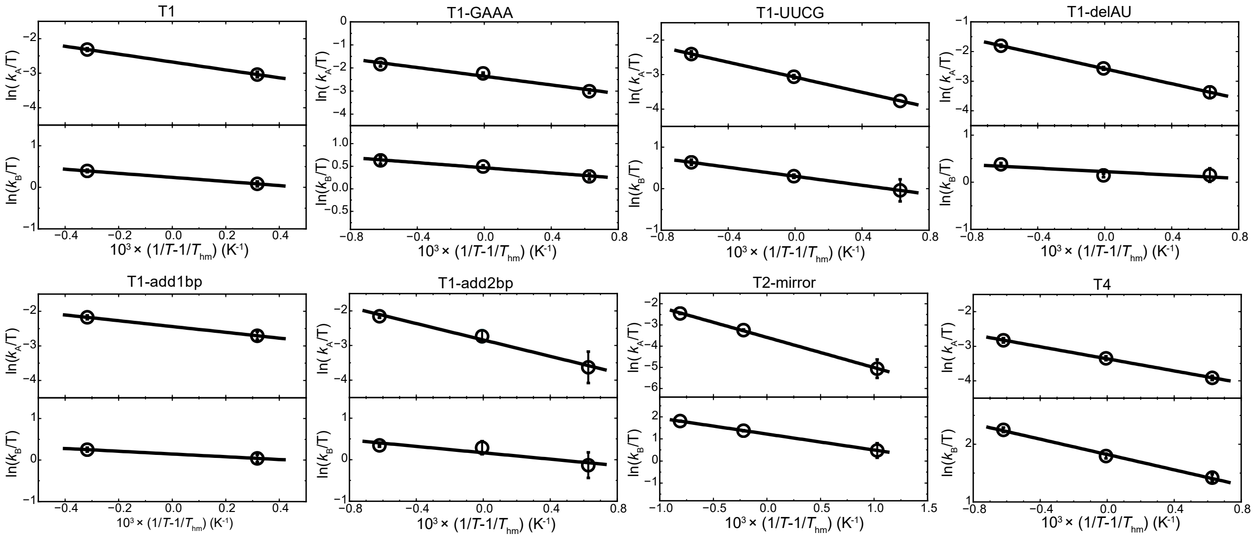

Supplementary Figure 15. Modified van't Hoff plots showing the temperature dependence of the forward (k_A) and reverse (k_B) rate constants for the two-site exchange processes of eight different RNAs. Error bars represent standard deviations (SD) as determined from the propagation of errors obtained from the fitting of $R_{1\rho}$ data (Monte Carlo simulation with 50 iterations; see Methods).

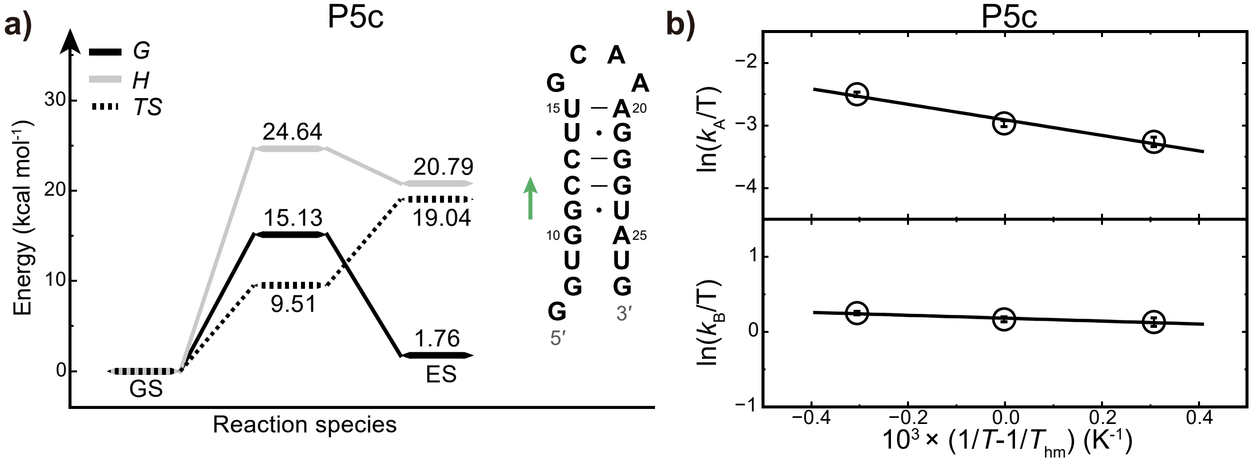

Supplementary Figure 16. (a) The energy diagram for the exchange process of P5c between GS and ES, in which the activation and net free energy (*G*), enthalpy (*H*), and entropy (*TS*) changes are shown. **(b)** Semi-logarithmic van't Hoff plots showing the dependence of the forward and backward rate constants on the temperature for P5c. Error bars represent standard deviations (SD) as determined from the propagation of errors obtained from the fitting of $R_{1\rho}$ data (Monte Carlo simulation with 50 iterations; see Methods).

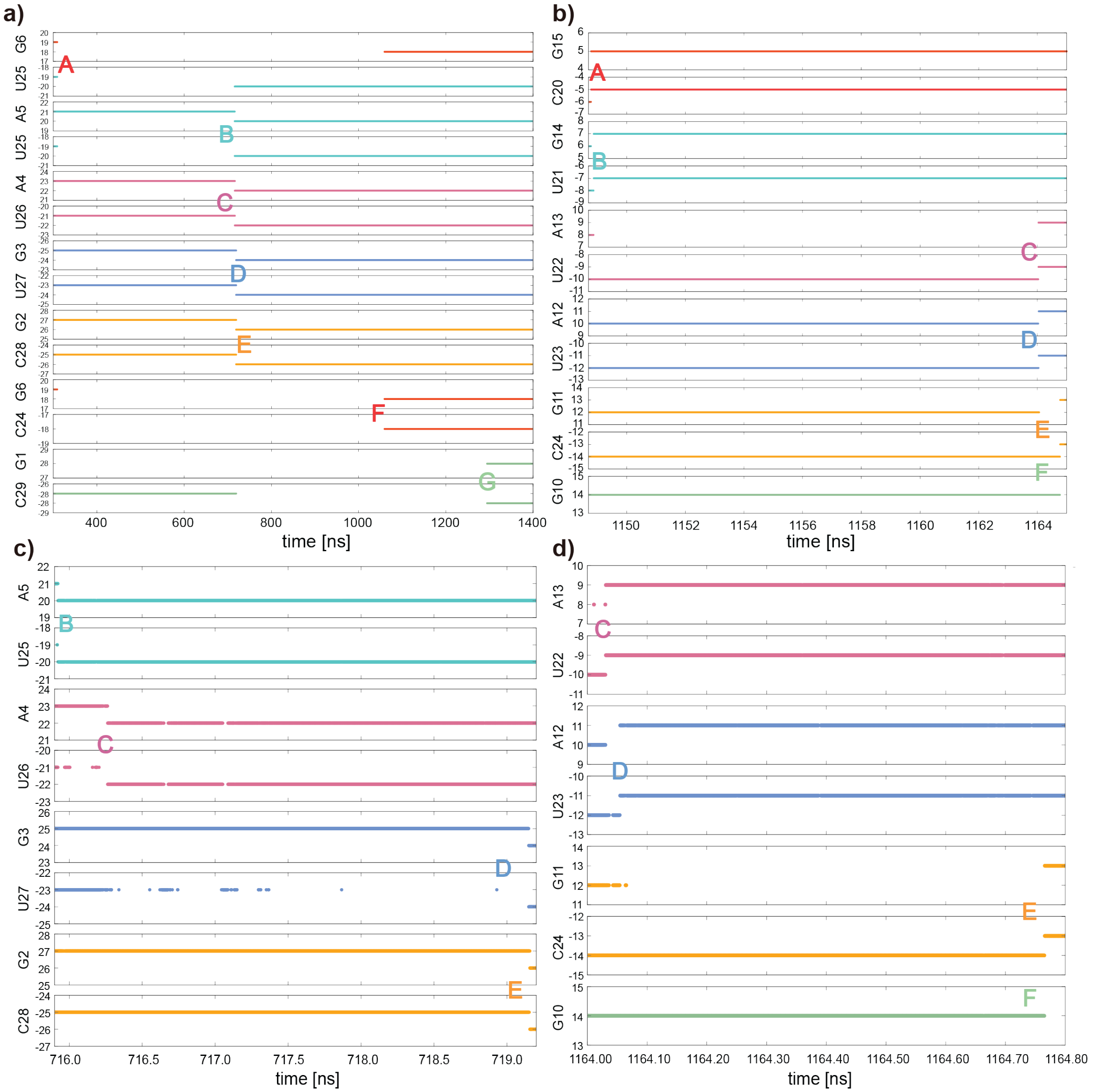

Supplementary Figure 17. The time courses of the entire transition events for T4 and T1 RNAs. **(a)** An ES-to-GS transition event for T4 RNA in a downward manner. **(b)** An ES-to-GS transition event for T1 RNA in a downward manner. **(c,d)** The enlarged time courses showing transition details for T4 and T1, respectively. The horizontal axis indicates the time of the aMD simulation, and the vertical axis represents the residue number difference between a residue of interest (shown to the left of y-axis) and another residue that pairs with it. When the residue of interest is unpaired, the residue number difference is set to zero. The lacapital letters represent time points for state transition, and use the same color scheme as in Figure 6.

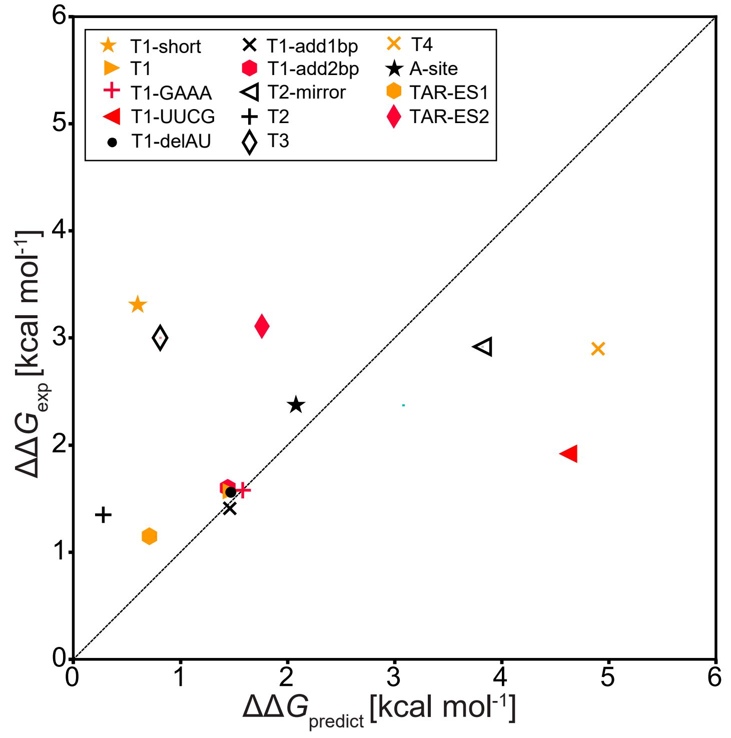

Supplementary Figure 18. Correlation between the measured free energy difference between the GS and ES by NMR RD experiments and the corresponding energy difference predicted by *MC-Fold*. The experimental $\Delta\Delta G$ for A-site and TAR-ESs were obtained from the published work[8-9].

Supplementary Tables

Supplementary Table 1. RNA sequences of all designed RNAs, and the corresponding populations of ESs (p_B) and the experimental free energy differences between GS and ES ($\Delta\Delta G$) at 10 °C.

RNA	Sequence	p_B (%)	k_1 (s^{-1})	k_{-1} (s^{-1})	$\Delta\Delta G$ (kcal mol^{-1})
T1-short	GGGAAGGGCAACUUUCA	0.28	5.6	1990	3.31
T1	GGCGCGAAAGGAAGGGCAACUUUCAAAACGCGCC	6.19	28	425	1.57
T1-GAAA	GGCGCGAAAGGAAGGGAAACUUUCAAAACGCGCC	6.08	31	473	1.58
T1-UUCG	GGCGCGAAAGGAAGGUUCGCUUUCAAAACGCGCC	3.27	13	393	1.92
T1-delAU	GGCGCGAAAGGAGGGCAACUUCAAAACGCGCC	6.25	22	325	1.56
T1-add1bp	GGCGCGAAAGGGAAGGGCAACUUUCUAAAACGCGCC	8.17	32	361	1.41
T1-add2bp	GGCGCGAAAGGGGAAGGGCAACUUUUCUAAAACGCGCC	5.78	20	331	1.60
T2-mirror	GGCGCGAAAACUUUCGCAAGGAAGGAAACGCGCC	0.56	3.2	577	2.92
T2	GGCGCGAAAGUUCCUGCAAAGGGAAAAACGCGCC	9.14	39	387	1.35
T3	GGCGCGAAAGGGUAUGGCAAUGUACAAAACGCGCC	0.50	7.2	1428	3.00
T4	GGGAAGUGCGCGCUUCGGCGCGCCUUUCC	0.58	10	1706	2.90

Supplementary Table 2. Acquisition parameters and fitting results of RD experiments for T1-short RNA. The upper table shows spin lock powers ($\omega_{SL}/2\pi$) and offsets ($\Omega/2\pi$) used in ^{15}N $R_{1\rho}$ RD experiments. The lower table shows the exchange parameters of T1-short obtained from individual and global fit of ^{15}N $R_{1\rho}$ data.

**Residue**	***T*(°C)**	**$\omega_{SL}/2\pi$ [Hz] & {$\Omega/2\pi$ [Hz]}**
G14-N1<br>U21-N3	10.0	100 & {±500,450,400,350,±300,250,±200,150,±100,50}<br>250 & {±800,±600,500,450,±400,350,300,±200,±100}<br>400 & {±1200,1000,±800,600,±500,450,400,350,±300,200,±100}

**Residue**	**R_2 (s^{-1})**	**k_{ex} (s^{-1})**	**p_B (%)**	**k_{ex} (s^{-1})**	**p_B (%)**	**$\bar{\omega}_{GS}$ [ppm]**	**$\Delta\bar{\omega}$ [ppm]**	**$\bar{\omega}_{ES}{}^{exptl}$ [ppm]**	**$\bar{\omega}_{ES}{}^{pred}$ [ppm]**
G14-N1	7.18±0.52	1596±194	0.39±0.02	1996±447	0.28±0.02	143.72	7.20±0.38	150.92	N/A
U21-N3	6.41±0.19	1554±294	0.23±0.02			158.19	5.48±0.39	163.67	162.67

Supplementary Table 3. Acquisition parameters and fitting results of RD experiments for T1 RNA. The upper table shows the spin-lock powers ($\omega_{SL}/2\pi$) and offsets ($\Omega/2\pi$) used in the ^{15}N $R_{1\rho}$ experiment at varying temperatures. The middle table lists the exchange parameters obtained by individually and globally fitting ^{15}N $R_{1\rho}$ and ^{1}H^{N} CEST data to a two-state model, as well as the experimental and predicted chemical shifts of ES at 10.0 °C. The lower table shows the exchange parameters of T1 obtained from individual and global fit of ^{15}N $R_{1\rho}$ data at different temperatures.

Residue	*T*(°C)	$\omega_{SL}/2\pi$ [Hz] & {$\Omega/2\pi$ [Hz]}
G14-N1<br>U21-N3	5.0<br>&<br>10.0	100 & {450,400,350,325,280,250,200,175,150,120,85,±50,15,-20,-100}<br>150 & {500,±400,350,300,250,±200,150,±100,75,±50}<br>200 & {500,±400,350,300,250,±200,150,±100,75,±50}<br>300 & {700,±500,400,350,±300,250,200,150,±100,±50}
G11-N1<br>G15-N1<br>U23-N3	10.0	100 & {100,±50,20,-15,-85,-120,-150,-175,-200,-250,-280,-325,-350,-400,-450}<br>150 & {±400,±200,±100,±50,-75,-150,-250,-300,-350,-500}<br>200 & {±400,±200,±100,±50,-75,-150,-250,-300,-350,-500}<br>300 & {±500,±300,±100,±50,-150,-200,-250,-350,-400,-700}
G16-N1	10.0	100 & {±400,350,±300,250,±200,±150,±100,±50}<br>150 & {500,±400,350,300,250,±200,150,±100,75,±50}<br>200 & {500,±400,350,300,250,±200,150,±100,75,±50}

Residue	R_2 (s^{-1})	k_{ex} (s^{-1})	p_B (%)	k_{ex} (s^{-1})	p_B (%)	$\bar{\omega}_{GS}$ [ppm]	$\Delta\bar{\omega}$ [ppm]	$\bar{\omega}_{ES}{}^{exptl}$ [ppm]	$\bar{\omega}_{ES}{}^{pred}$ [ppm]
G11-N1	13.18±0.28	420±44	5.7±0.5			146.42	-3.94±0.07	142.48	143.15
G11-H1	fixed*	fixed*	fixed*			11.78	-1.32±0.01	10.46	10.70
G14-N1	13.09±0.31	442±23	6.5±0.3			143.34	4.71±0.06	148.05	N/A
G14-H1	fixed*	fixed*	fixed*			10.97	2.35±0.01	13.32	N/A
G15-N1	13.33±0.10	771±120	14.5±12.4			148.02	-1.03±0.03	146.99	N/A
G15-H1	fixed*	fixed*	fixed*			13.31	-2.46±0.01	10.85	N/A
G16-N1	12.50±0.29	528±197	5.4±3.2	453±13	6.2±0.1	146.25	1.13±0.06	147.38	N/A
G16-H1	fixed*	fixed*	fixed*			10.92	0.07±0.03	10.99	N/A
U21-N3	12.31±0.22	441±16	6.5±0.2			158.09	4.95±0.04	163.04	162.67
U21-H3	fixed*	fixed*	fixed*			11.94	2.13±0.01	14.07	14.19
U22-N3	12.24±0.20	No RD	No RD			162.77	0.00±0.00	162.77	162.52
U22-H3	fixed*	fixed*	fixed*			14.37	-0.59±0.01	13.78	13.85
U23-N3	12.05±0.44	452±16	6.1±0.2			163.32	-5.55±0.06	157.77	158.75
U23-H3	fixed*	fixed*	fixed*			14.07	-2.31±0.01	11.76	12.08

* R_2 can not be well defined by fitting the CEST profile, and thus was fixed to a typical value (25 s^{-1}). The parameters of H1/H3 k_{ex} and p_B were fixed to the globally fitting results.

Residue	R_2 (s^{-1})	*T* (°C)	k_{ex} (s^{-1})	p_B (%)	k_{ex} (s^{-1})	p_B (%)	$\Delta\bar{\omega}$ [ppm]
G14-N1	15.50±0.17	5.0	334±24	4.1±0.2	316±18	4.2±0.2	4.53±0.07
U21-N3	14.25±0.14		307±18	4.3±0.2			4.81±0.04
G14-N1	13.09±0.31	10.0	442±23	6.5±0.3	453±13	6.2±0.1	4.71±0.06
U21-N3	12.31±0.22		441±16	6.5±0.2			4.95±0.04

Supplementary Table 4. Acquisition parameters and fitting results of RD experiments for T1-GAAA. The upper table shows the spin-lock powers ($\omega_{SL}/2\pi$) and offsets ($\Omega/2\pi$) used in the ^{15}N $R_{1\rho}$ experiment at varying temperatures. The middle table lists the exchange parameters obtained by individually and globally fitting ^{15}N $R_{1\rho}$ and $^{1}H^{N}$ CEST data to a two-state model, as well as the experimental and predicted chemical shifts of ES at 10 °C. The lower table shows the exchange parameters of T1-GAAA obtained from individual and global fit of ^{15}N $R_{1\rho}$ data at different temperatures.

Residue	*T* (°C)	$\omega_{SL}/2\pi$ [Hz] & {$\Omega/2\pi$ [Hz]}
**G14-N1**<br>**U21-N3**	**5.0**<br>**&**<br>**10.0**	100 & {450,400,350,325,280,250,200,175,150,120,85,±50,15,-20,-100}<br>150 & {500,±400,350,300,250,±200,150,±100,75,±50}<br>200 & {500,±400,350,300,250,±200,150,±100,75,±50}<br>300 & {700,±500,400,350,±300,250,200,150,±100,±50}
	**15.0**	100 & {450,400,350,325,280,250,200,175,150,120,85,±50,15,-20,-100}<br>150 & {500,±400,350,300,250,±200,150,±100,±50}<br>200 & {500,±400,350,300,250,±200,150,±100,±50}<br>300 & {700,±500,400,350,±300,250,200,150,±100,±50}
**G11-N1**<br>**G15-N1**<br>**U23-N3**	**10.0**	100 & {100,±50,20,-15,-85,-120,-150,-175,-200,-250,-280,-325,-350,-400,-450}<br>150 & {±400,±200,±100,±50,-75,-150,-250,-300,-350,-500}<br>200 & {±400,±200,±100,±50,-75,-150,-250,-300,-350,-500}<br>300 & {±500,±300,±100,±50,-150,-200,-250,-350,-400,-700}
**G16-N1**	**10.0**	100 & {±400,350,±300,250,±200,±150,±100,±50}<br>150 & {500,±400,350,300,250,±200,150,±100,75,±50}<br>200 & {500,±400,350,300,250,±200,150,±100,75,±50}

Residue	R_2 (s^{-1})	k_{ex} (s^{-1})	p_B (%)	k_{ex} (s^{-1})	p_B (%)	$\bar{\omega}_{GS}$ [ppm]	$\Delta\bar{\omega}$ [ppm]	$\bar{\omega}_{ES}^{exptl}$ [ppm]	$\bar{\omega}_{ES}^{pred}$ [ppm]
G11-N1	13.46±0.30	499±28	6.1±0.3	504±13	6.1±0.1	146.42	-4.09±0.06	142.33	143.15
G11-H1	fixed*	fixed*	fixed*			11.79	-1.31±0.01	10.48	10.70
G14-N1	12.81±0.23	497±19	6.2±0.2			143.38	4.68±0.05	148.06	N/A
G14-H1	fixed*	fixed*	fixed*			10.98	2.32±0.01	13.30	N/A
G15-N1	13.52±0.11	884±39	38.4±10.4			147.99	-1.26±0.02	146.73	N/A
G15-H1	fixed*	fixed*	fixed*			13.29	-2.58±0.01	10.71	N/A
G16-N1	12.44±0.17	575±131	7.5±3.5			146.14	1.01±0.04	147.15	N/A
G16-H1	fixed*	fixed*	fixed*			10.92	-0.08±0.01	10.84	N/A
U21-N3	12.20±0.18	489±14	6.2±0.2			158.08	4.88±0.03	162.96	162.67
U21-H3	fixed*	fixed*	fixed*			11.92	2.15±0.01	14.07	14.19
U22-N3	12.41±0.14	No RD	No RD			162.78	0.00±0.00	162.78	162.52
U22-H3	fixed*	fixed*	fixed*			14.37	-0.56±0.01	13.81	13.85
U23-N3	12.26±0.29	499±19	6.1±0.2			163.29	-5.34±0.07	157.95	158.75
U23-H3	fixed*	fixed*	fixed*			14.08	-2.30±0.01	11.78	12.08

* R_2 can not be well defined by fitting the CEST profile, and thus was fixed to a typical value (25 s^{-1}). The parameters of H1/H3 k_{ex} and p_B were fixed to the globally fitting results.

Residue	R_2 (s^{-1})	*T* (°C)	k_{ex} (s^{-1})	p_B (%)	k_{ex} (s^{-1})	p_B (%)	$\Delta\bar{\omega}$ [ppm]
G14-N1	15.37±0.16	5.0	393±28	3.5±0.2	380±24	3.6±0.2	4.58±0.05
U21-N3	14.47±0.17		370±26	3.7±0.2			4.85±0.07
G14-N1	12.81±0.23	10.0	497±19	6.2±0.2	504±13	6.1±0.1	4.68±0.05
U21-N3	12.20±0.18		489±14	6.2±0.2			4.88±0.03
G14-N1	12.31±0.82	15.0	607±50	7.8±0.6	587±44	7.8±0.5	4.97±0.13
U21-N3	12.23±0.70		574±39	7.9±0.4			5.12±0.10

Supplementary Table 5. Acquisition parameters and fitting results of RD experiments for T1-UUCG. The upper table shows the spin-lock powers ($\omega_{SL}/2\pi$) and offsets ($\Omega/2\pi$) used in the ^{15}N $R_{1\rho}$ experiment at varying temperatures. The middle table lists the exchange parameters obtained by individually and globally fitting ^{15}N $R_{1\rho}$ and ^{1}H^{N} CEST data to a two-state model, as well as the experimental and predicted chemical shifts of ES at 10 °C. The lower table shows the exchange parameters of T1-UUCG obtained from individual and global fit of ^{15}N $R_{1\rho}$ data at different temperatures.

Residue	*T* (°C)	$\omega_{SL}/2\pi$ [Hz] & {$\Omega/2\pi$ [Hz]}
U21-N3	5.0 & 10.0 (G14, G19 (N1))	100 & {400,350,325,300,250,200,175,150,125,±100,75,±50,±25}
		150 & {500,±400,350,300,250,±200,175,150,125,±100,75,±50}
		200 & {500,±400,350,300,250,±200,175,150,125,±100,75,±50}
		300 & {700,±500,400,350,±300,250,200,175,150,125,±100,±50}
	15.0 (G14 (N1))	100 & {400,350,±300,250,±200,150,±100,50}
		150 & {550,450,350,±300,250,200,±150,100,±50}
		200 & {700,500,400,350,±300,250,200,±150,100,50}
G11-N1 G15-N1 U23-N3	10.0	100 & {-400,-350,-325,-300,-250,-200,-175,-150,-125,±100,-75,±50,±25}
		150 & {-500,±400,-350,-300,-250,±200,-175,-150,-125,±100,-75,±50}
		200 & {-500,±400,-350,-300,-250,±200,-175,-150,-125,±100,-75,±50}
		300 & {-700,±500,-400,-350,±300,-250,-200,-175,-150,-125,±100,±50}

Residue	R_2 (s^{-1})	k_{ex} (s^{-1})	p_B (%)	k_{ex} (s^{-1})	p_B (%)	$\bar{\omega}_{GS}$ [ppm]	$\Delta\bar{\omega}$ [ppm]	$\bar{\omega}_{ES}^{exptl}$ [ppm]	$\bar{\omega}_{ES}^{pred}$ [ppm]
G11-N1	14.03±0.14	359±28	3.7±0.3	406±13	3.3±0.1	146.45	-3.96±0.06	142.49	143.15
G11-H1	fixed*	fixed*	fixed*			11.73	-1.32±0.01	10.41	10.70
G14-N1	13.52±0.13	443±24	3.1±0.1			143.57	4.86±0.05	148.43	N/A
G14-H1	fixed*	fixed*	fixed*			11.02	2.30±0.01	13.32	N/A
G15-N1	12.86±0.11	390±72	2.7±0.4			148.48	-2.11±0.04	146.37	N/A
G15-H1	fixed*	fixed*	fixed*			13.86	-0.10±0.01	13.76	N/A
G19-N1	12.97±0.07	436±34	3.0±0.2			144.67	2.41±0.02	147.08	N/A
G19-H1	fixed*	fixed*	fixed*			10.50	-0.03±0.01	10.47	N/A
U21-N3	12.54±0.11	386±17	3.4±0.1			158.27	4.74±0.03	163.01	162.67
U21-H3	fixed*	fixed*	fixed*			11.97	2.33±0.01	14.30	14.19
U22-N3	10.31±0.19	No RD	No RD			162.81	0.00±0.00	162.81	162.52
U22-H3	fixed*	fixed*	fixed*			14.25	-0.56±0.01	13.69	13.85
U23-N3	12.59±0.16	395±25	3.3±0.2			163.32	-5.15±0.07	158.17	158.75
U23-H3	fixed*	fixed*	fixed*			14.03	-2.30±0.01	11.73	12.08

∗ R_2 can not be well defined by fitting the CEST profile, and thus was fixed to a typical value (25 s^{-1}). The parameters of H1/H3 k_{ex} and p_B were fixed to the globally fitting results.

Residue	R_2 (s^{-1})	*T* (°C)	k_{ex} (s^{-1})	p_B (%)	k_{ex} (s^{-1})	p_B (%)	$\Delta\bar{\omega}$ [ppm]
U21-N3	14.10±0.16	5.0	274±66	2.4±0.7	274±66	2.4±0.7	4.89±0.06
G14-N1	13.52±0.13	10.0	443±24	3.1±0.1	406±13	3.3±0.1	4.86±0.05
U21-N3	12.54±0.11		386±17	3.4±0.1			4.74±0.03
G14-N1	11.68±0.47	15.0	583±61	4.3±0.4	571±36	4.6±0.2	4.84±0.14
U21-N3	11.27±0.34		567±40	4.6±0.2			4.89±0.09

Supplementary Table 6. Acquisition parameters and fitting results of RD experiments for T1-delAU. The upper table shows the spin-lock powers ($\omega_{SL}/2\pi$) and offsets ($\Omega/2\pi$) used in the ^{15}N $R_{1\rho}$ experiment at varying temperatures. The middle table lists the exchange parameters obtained by individually and globally fitting ^{15}N $R_{1\rho}$ and ^{1}H^{N} CEST data to a two-state model, as well as the experimental and predicted chemical shifts of ES at 10 °C. The lower table shows the exchange parameters of T1-delAU obtained from individual and global fit of ^{15}N $R_{1\rho}$ data at different temperatures.

**Residue**	***T* (°C)**	**$\omega_{SL}/2\pi$ [Hz] & {$\Omega/2\pi$ [Hz]}**
**G13-N1**	**5.0(U20 (N3))<br>10.0(U20(N3))<br>&15.0**	100 & {400,350,325,300,250,200,175,150,125,±100,75,±50,±25}
		150 & {500,±400,350,300,250,±200,175,150,125,±100,75,±50}
		200 & {500,±400,350,300,250,±200,175,150,125,±100,75,±50}
		300 & {700,±500,400,350,±300,250,200,175,150,125,±100,±50}
**U20-N3**	**15.0**	100 & {450,400,350,325,280,250,200,175,150,120,-100,85,±50,15,-20}
		150 & {500,±400,350,300,250,±200,150,±100,75,±50}
		200 & {500,±400,350,300,250,±200,150,±100,75,±50}
		300 & {700,±500,400,350,±300,250,200,150,±100,±50}
**G11-N1**	**10.0**	100 & {100,±50,±25,-75,-100,-125,-150,-175,-200,-250,-300,-325,-350,-400}
		150 & {±400,±200,±100,±50,-75,-125,-150,-175,-250,-300,-350,-500}
		200 & {±400,±200,±100,±50,-75,-125,-150,-175,-250,-300,-350,-500}
		300 & {±500,±300,±100,±50,-125,-150,-175,-200,-250,-350,-400,-700}
**G14-N1**	**10.0**	100 & {±400,350,±300,250,±200,±150,±100,±50}
		150 & {500,±400,350,300,250,±200,±150,±100,±50}
		200 & {500,±400,350,300,250,±200,±150,±100,±50}
**G15-N1**	**10.0**	100 & {±400,350,±300,250,±200,±150,±100,±50}
		150 & {500,±400,350,300,250,±200,150,±100,75,±50}
		200 & {500,±400,350,300,250,±200,150,±100,75,±50}
**U21-N3**	**10.0**	100 & {100,±50,20,-15,-85,-120,-150,-175,-200,-250,-280,-325,-350,-400,-450}
		150 & {±400,±200,±100,±50,-75,-150,-250,-300,-350,-500}
		200 & {±400,±200,±100,±50,-75,-150,-250,-300,-350,-500}
		300 & {±500,±300,±100,±50,-150,-200,-250,-350,-400,-700}

**Residue**	***R_2* (s^{-1})**	***k_{ex}* (s^{-1})**	***p_B* (%)**	***k_{ex}* (s^{-1})**	***p_B* (%)**	**$\bar{\omega}_{GS}$ [ppm]**	**$\Delta\bar{\omega}$ [ppm]**	**$\bar{\omega}_{ES}{}^{exptl}$ [ppm]**	**$\bar{\omega}_{ES}{}^{pred}$ [ppm]**
G11-N1	13.86±0.28	334±18	6.3±0.3			146.28	-3.92±0.08	142.36	143.15
G11-H1	fixed*	fixed*	fixed*			11.85	-1.32±0.01	10.53	10.70
G13-N1	13.25±0.21	362±21	6.0±0.3			143.42	4.50±0.06	138.92	N/A
G13-H1	fixed*	fixed*	fixed*			11.02	2.32±0.01	13.34	N/A
G14-N1	12.87±0.21	442±165	27.1±10.2			148.05	1.24±0.05	149.29	N/A
G14-H1	fixed*	fixed*	fixed*	347±10	6.3±0.2	13.36	-2.44±0.01	10.92	N/A
G15-N1	12.22±0.22	253±171	7.6±6.4			146.25	1.08±0.07	147.33	N/A
G15-H1	fixed*	fixed*	fixed*			10.97	-0.06±0.01	10.91	N/A
U20-N3	12.62±0.20	337±16	6.5±0.2			158.16	4.10±0.05	162.26	162.22
U20-H3	fixed*	fixed*	fixed*			11.96	2.06±0.01	14.02	14.15
U21-N3	12.49±0.32	360±14	6.1±0.2			163.45	-5.61±0.06	157.84	158.75
U21-H3	fixed*	fixed*	fixed*			14.67	-2.87±0.01	11.80	12.08

* R_2 can not be well defined by fitting the CEST profile, and thus was fixed to a typical value (25 s^{-1}). The parameters of H1/H3 k_{ex} and p_B were fixed to the globally fitting results.

**Residue**	***R_2* (s^{-1})**	***T* (°C)**	***k_{ex}* (s^{-1})**	***p_B* (%)**	***k_{ex}* (s^{-1})**	***p_B* (%)**	**$\Delta\bar{\omega}$ [ppm]**
G13-N1	15.72±0.21	5.0	352±44	2.7±0.3	333±47	2.9±0.3	4.32±0.11
U20-N3	14.84±0.20		314±53	3.0±0.4			3.95±0.12
G13-N1	13.25±0.21	10.0	362±21	6.0±0.3	347±10	6.3±0.2	4.50±0.06
U20-N3	12.62±0.20		337±16	6.5±0.2			4.10±0.05
G13-N1	10.19±0.57	15.0	487±18	9.9±0.3	469±14	10.1±0.3	4.74±0.08
U20-N3	10.51±0.57		451±15	10.4±0.3			4.27±0.08

Supplementary Table 7. Acquisition parameters and fitting results of RD experiments for T1-add1bp. The upper table shows the spin-lock powers ($\omega_{SL}/2\pi$) and offsets ($\Omega/2\pi$) used in the ^{15}N $R_{1\rho}$ experiment at varying temperatures. The middle table lists the exchange parameters obtained by individually and globally fitting ^{15}N $R_{1\rho}$ and ^{1}H^{N} CEST data to a two-state model, as well as the experimental and predicted chemical shifts of ES at 10 °C. The lower table shows the exchange parameters of T1-add1bp obtained from individual and global fit of ^{15}N $R_{1\rho}$ data at different temperatures.

Residue	T (°C)	$\omega_{SL}/2\pi$ [Hz] & {$\Omega/2\pi$ [Hz]}
G12-N1	10.0	100 & {-400,-350,-325,±300,-250,-225,±200,-175,-150,±100,-50}<br>150 & {-550,-450,-400,-350,±300,-250,-200,±150,-100,-50}<br>250 & {-700,-600,-500,-400,-350,±300,-250,-200,±150,-100,-50}
G15-N1	5.0 & 10.0	100 & {400,350,±300,250,±200,150,±100,50}<br>150 & {550,450,350,±300,250,200,±150,100,50}<br>250 & {700,500,400,350,±300,250,200,±150,100,50}
G16-N1<br>G17-N1	10.0	100 & {±400,350,±300,250,±200,150,±100,75,±50}<br>150 & {500,±400,350,300,250,±200,150,±100,75,±50}<br>200 & {500,±400,350,300,250,±200,150,±100,75,±50}
U22-N3	5.0 & 10.0	100 & {400,350,325,±300,250,±200,150,±100,50}<br>150 & {550,450,400,350,±300,250,200,±150,100,50}<br>250 & {700,500,400,350,±300,250,200,±150,100,50}
U24-N3	10.0	100 & {-400,-350,-325,±300,-250,±200,-150,±100,-50}<br>150 & {-550,-450,-400,-350,±300,-250,-200,±150,-100,-50}<br>250 & {-700,-500,-400,-350,±300,-250,-200,±150,-100,-50}

Residue	R_2 (s^{-1})	k_{ex} (s^{-1})	p_B (%)	k_{ex} (s^{-1})	p_B (%)	$\bar{\omega}_{GS}$ [ppm]	$\Delta\bar{\omega}$ [ppm]	$\bar{\omega}_{ES}^{exptl}$ [ppm]	$\bar{\omega}_{ES}^{pred}$ [ppm]
G12-N1	14.93±0.84	337±52	8.0±1.0	393±18	8.2±0.3	147.07	-4.56±0.16	142.51	143.15
G12-H1	fixed*	fixed*	fixed*			12.53	-1.93±0.01	10.60	10.70
G15-N1	16.92±0.51	398±24	8.8±0.4			143.60	4.79±0.11	148.39	N/A
G15-H1	fixed*	fixed*	fixed*			11.10	2.33±0.01	13.43	N/A
G16-N1	14.48±0.31	367±253	13.3±12.7			148.20	1.19±0.06	149.39	N/A
G16-H1	fixed*	fixed*	fixed*			13.44	-2.46±0.01	10.98	N/A
G17-N1	13.69±0.53	118±176	15.2±6.8			146.28	1.13±0.11	147.41	N/A
G17-H1	fixed*	fixed*	fixed*			11.04	-0.07±0.01	10.97	N/A
U22-N3	15.70±0.53	404±17	8.9±0.3			158.26	5.04±0.07	163.30	162.67
U22-H3	fixed*	fixed*	fixed*			12.05	2.13±0.01	14.18	14.19
U23-N3	13.38±0.39	No RD	No RD			162.88	0.00±0.00	162.88	162.52
U23-H3	fixed*	fixed*	fixed*			14.42	-0.54±0.01	13.88	13.85
U24-N3	15.08±0.57	397±27	7.3±0.4			163.13	-5.24±0.09	157.89	158.75
U24-H3	fixed*	fixed*	fixed*			13.93	-2.03±0.01	11.90	12.08

* R_2 can not be well defined by fitting the CEST profile, and thus was fixed to a typical value (25 s^{-1}). The parameters of H1/H3 k_{ex} and p_B were fixed to the globally fitting results.

Residue	R_2 (s^{-1})	T (°C)	k_{ex} (s^{-1})	p_B (%)	k_{ex} (s^{-1})	p_B (%)	$\Delta\bar{\omega}$ [ppm]
G15-N1	19.24±0.52	5.0	320±30	5.9±0.5	308±28	6.0±0.4	4.57±0.13
U22-N3	18.17±0.36		300±27	6.2±0.5			4.86±0.10
G15-N1	16.92±0.51	10.0	398±24	8.8±0.4	393±18	8.2±0.3	4.79±0.11
U22-N3	15.70±0.53		404±17	8.9±0.3			5.04±0.07

Supplementary Table 8. Acquisition parameters and fitting results of RD experiments for T1-add2bp. The upper table shows the spin-lock powers ($\omega_{SL}/2\pi$) and offsets ($\Omega/2\pi$) used in the ^{15}N $R_{1\rho}$ experiment at varying temperatures. The middle table lists the exchange parameters obtained by individually and globally fitting ^{15}N $R_{1\rho}$ and ^{1}H^{N} CEST data to a two-state model, as well as the experimental and predicted chemical shifts of ES at 10 °C. The lower table shows the exchange parameters of T1-add2bp obtained from individual and global fit of ^{15}N $R_{1\rho}$ data at different temperatures.

Residue	*T* (°C)	$\omega_{SL}/2\pi$ [Hz] & {$\Omega/2\pi$ [Hz]}
G12-N1	10.0	100 & {-400,-350,-325,-300,-250,-200,-175,-150,-125,±100,-75,±50,±25}<br>150 & {-500,±400,-350,-300,-250,±200,-175,-150,-125,±100,-75,±50}<br>200 & {-500,±400,-350,-300,-250,±200,-175,-150,-125,±100,-75,±50}<br>300 & {-700,±500,-400,-350,±300,-250,-200,-175,-150,-125,±100,±50}
G16-N1	10.0	100 & {450,400,350,325,280,250,200,175,150,120,-100,85,±50,-20,15}<br>150 & {500,±400,350,300,250,±200,150,±100,75,±50}<br>200 & {500,±400,350,300,250,±200,150,±100,75,±50}<br>300 & {700,±500,400,350,±300,250,200,150,±100,±50}
G16-N1	5.0<br>&<br>15.0	100 & {400,350,325,300,250,200,175,150,125,±100,75,±50,±25}<br>150 & {500,±400,350,300,250,±200,175,150,125,±100,75,±50}<br>200 & {500,±400,350,300,250,±200,175,150,125,±100,75,±50}<br>300 & {700,±500,400,350,±300,250,200,175,150,125,±100,±50}
U23-N3	10.0	100 & {450,400,350,325,280,250,200,175,150,120,85,±50,-20,15}<br>150 & {500,±400,350,300,250,±200,150,±100,75,±50}<br>200 & {500,±400,350,300,250,±200,150,±100,75,±50}<br>300 & {700,±500,400,350,±300,250,200,150,±100,±50}
U23-N3	5.0	100 & {400,350,325,300,250,200,175,150,125,±100,75,±50,±25}<br>150 & {500,±400,350,300,250,±200,175,150,125,±100,75,±50}<br>200 & {500,±400,350,300,250,±200,175,150,125,±100,75,±50}<br>300 & {700,±500,400,350,±300,250,200,175,150,125,±100,±50}
U25-N3	5.0<br>&<br>15.0	100 & {-400,-350,-325,-300,-250,-200,-175,-150,-125,±100,-75,±50,±25}<br>150 & {-500,±400,-350,-300,-250,±200,-175,-150,-125,±100,-75,±50}<br>250 & {-500,±400,-350,-300,-250,±200,-175,-150,-125,±100,-75,±50}<br>300 & {-700,±500,-400,-350,±300,-250,-200,-175,-150,-125,±100,±50}

Residue	R_2 (s^{-1})	k_{ex} (s^{-1})	p_B (%)	k_{ex} (s^{-1})	p_B (%)	$\bar{\omega}_{GS}$ [ppm]	$\Delta\bar{\omega}$ [ppm]	$\bar{\omega}_{ES}^{exptl}$ [ppm]	$\bar{\omega}_{ES}^{pred}$ [ppm]
G12-N1	18.11±0.29	334±26	6.2±0.4			147.97	-4.69±0.07	143.28	143.72
G12-H1	fixed*	fixed*	fixed*			12.85	-1.95±0.01	10.90	10.89
G16-N1	16.32±0.20	366±11	5.8±0.2			143.77	4.56±0.04	148.33	N/A
G16-H1	fixed*	fixed*	fixed*			11.15	2.33±0.01	13.48	N/A
G17-N1	15.40±0.24	801±207	15.0±22.2	351±11	5.8±0.2	148.53	-1.21±0.05	147.32	N/A
G17-H1	fixed*	fixed*	fixed*			13.49	-2.45±0.01	11.04	N/A
U24-N1	13.93±0.19	959±584	0.8±3.0			163.04	-0.76±0.08	162.28	162.52
U24-H1	fixed*	fixed*	fixed*			14.46	-0.63±0.01	13.83	13.85
U25-N3	14.59±0.15	343±13	5.7±0.2			162.68	-4.94±0.05	157.74	158.45
U25-H3	fixed*	fixed*	fixed*			13.94	-2.13±0.01	11.81	11.89

∗ R_2 can not be well defined by fitting the CEST profile, and thus was fixed to a typical value (25 s^{-1}). The parameters of H1/H3 k_{ex} and p_B were fixed to the globally fitting results.

Residue	R_2 (s^{-1})	*T* (°C)	k_{ex} (s^{-1})	p_B (%)	k_{ex} (s^{-1})	p_B (%)	$\Delta\bar{\omega}$ [ppm]
G16-N1	19.99±0.19	5.0	272±50	3.4±0.6	272±50 s^{-1}	3.4±0.6 %	4.54±0.11
G16-N1	16.32±0.20	10.0	366±11	5.8±0.2	351±11 s^{-1}	5.8±0.2 %	4.56±0.04
U25-N3	14.59±0.15		343±13	5.7±0.2			-4.94±0.05
G16-N1	14.37±0.41	15.0	438±19	9.5±0.3	440±14 s^{-1}	7.6±0.2 %	4.95±0.07
U25-N3	12.45±0.31		440±12	7.2±0.2			-5.03±0.06

Supplementary Table 9. Acquisition parameters and fitting results of RD experiments for T2-mirror. The upper table shows the spin-lock powers ($\omega_{SL}/2\pi$) and offsets ($\Omega/2\pi$) used in the ^{15}N $R_{1\rho}$ experiment at varying temperatures. The middle table lists the exchange parameters obtained by individually and globally fitting ^{15}N $R_{1\rho}$ and $^{1}H^{N}$ CEST data to a two-state model, as well as the experimental and predicted chemical shifts of ES at 10 °C. The lower table shows the exchange parameters of T1-mirror obtained from individual and global fit of ^{15}N $R_{1\rho}$ data at different temperatures.

Residue	T (°C)	$\omega_{SL}/2\pi$ [Hz] & {$\Omega/2\pi$ [Hz]}
**G21-N1<br>U14-N3**	**10.0**	100 & {400,350,325,300,250,200,175,150,125,±100,75,±50,±25}<br>150 & {500,±400,350,300,250,±200,175,150,125,±100,75,±50}<br>200 & {500,±400,350,300,250,±200,175,150,125,±100,75,±50}<br>300 & {700,±500,400,350,±300,250,200,175,150,125,±100,±50}
**G24-N1**	**10.0**	100 & {-400,-350,-325,-300,-250,-200,-175,-150,-125,±100,-75,±50,±25}<br>150 & {-500,±400,-350,-300,-250,±200,-175,-150,-125,±100,-75,±50}<br>200 & {-500,±400,-350,-300,-250,±200,-175,-150,-125,±100,-75,±50}<br>300 & {-700,±500,-400,-350,±300,-250,-200,-175,-150,-125,±100,±50}
**G21-N1<br>U14-N3**	**5.0&<br>15.0&<br>20.0**	150 & {500,400,350,300,250,200,175,±150,125,100,±75,50,±25}<br>200 & {500,400,350,300,250,200,175,±150,125,100,±75,50,±25}<br>300 & {700,±500,400,350,±300,250,200,175,150,125,±100,±50}
**G24-N1**	**20.0**	150 & {-500,-400,-350,-300,-250,-200,-175,±150,-125,-100,±75,-50,±25}<br>200 & {-500,-400,-350,-300,-250,-200,-175,±150,-125,-100,±75,-50,±25}<br>300 & {-700,±500,-400,-350,±300,-250,-200,-175,-150,-125,±100,±50}

Residue	R_2 (s^{-1})	k_{ex} (s^{-1})	p_B (%)	k_{ex} (s^{-1})	p_B (%)	$\bar{\omega}_{GS}$ [ppm]	$\Delta\bar{\omega}$ [ppm]	$\bar{\omega}_{ES}{}^{exptl}$ [ppm]	$\bar{\omega}_{ES}{}^{pred}$ [ppm]
U14-N3	12.41±0.05	512±33	0.59±0.03			158.65	4.90±0.07	163.55	163.15
U14-H3	fixed*	fixed*	fixed*			11.98	2.23±0.01	14.21	13.98
G21-N1	13.20±0.08	670±53	0.59±0.02	580±39	0.56±0.03	142.97	4.47±0.11	147.44	N/A
G21-H1	fixed*	fixed*	fixed*			10.70	1.70±0.01	12.40	N/A
G24-N1	13.86±0.10	802±118	0.42±0.03			148.59	-4.53±0.26	144.06	143.49
G24-H1	fixed*	fixed*	fixed*			13.44	-2.17±0.03	11.27	11.11

* R_2 can not be well defined by fitting the CEST profile, and thus was fixed to a typical value (25 s^{-1}). The parameters of H1/H3 k_{ex} and p_B were fixed to the globally fitting results.

Residue	R_2 (s^{-1})	T (°C)	k_{ex} (s^{-1})	p_B (%)	k_{ex} (s^{-1})	p_B (%)	$\Delta\bar{\omega}$ [ppm]
U14-N3	16.13±0.06	5.0	349±180	0.45±0.44	451±132	0.39±0.13	6.85±0.18
G21-N1	17.53±0.10		604±129	0.32±0.55			6.27±0.26
U14-N3	12.08±0.10	15.0	1152±30	0.98±0.01	1151±26	0.98±0.01	6.56±0.09
G21-N1	13.02±0.09		1155±35	1.00±0.02			6.08±0.09
U14-N1	11.35±0.19		1690±49	1.33±0.02			6.84±0.10
G21-N1	11.84±0.17	20.0	1843±52	1.39±0.04	1821±40	1.31±0.02	6.38±0.11
G24-N1	12.01±0.19		1990±88	1.07±0.03			5.43±0.11

Supplementary Table 10. Acquisition parameters and fitting results of RD experiments for T2 RNA. The upper table shows the spin-lock powers ($\omega_{SL}/2\pi$) and offsets ($\Omega/2\pi$) used in the ^{15}N $R_{1\rho}$ experiment at varying temperatures. The lower table lists the exchange parameters obtained by individually and globally fitting ^{15}N $R_{1\rho}$ and $^{1}H^{N}$ CEST data to a two-state model, as well as the experimental and predicted chemical shifts of ES at 10 °C.

Residue	T (°C)	$\omega_{SL}/2\pi$ [Hz] & {$\Omega/2\pi$ [Hz]}
G22-N1<br>U15-N3	10.0	100 & {-400,-350,-300,-250,±200,-150,±100,±50}<br>150 & {-550,-450,-350,±300,-250,-200,±150,-100,±50}<br>250 & {-700,-500,-400,-350,±300,-250,-200,±150,-100,±50}
G23-N1<br>U12-N3	10.0	100 & {450,400,350,325,280,250,200,175,150,120,-100,85,±50,-20,15}<br>150 & {500,±400,350,300,250,±200,150,±100,75,±50}<br>200 & {500,±400,350,300,250,±200,150,±100,75,±50}<br>300 & {700,±500,400,350,±300,250,200,150,±100,±50}

.

Residue	R_2 (s^{-1})	k_{ex} (s^{-1})	p_B (%)	k_{ex} (s^{-1})	p_B (%)	$\bar{\omega}_{GS}$ [ppm]	$\Delta\bar{\omega}$ [ppm]	$\bar{\omega}_{ES}{}^{exptl}$ [ppm]	$\bar{\omega}_{ES}{}^{pred}$ [ppm]
U12-N3	17.88±0.50	430±13	9.1±0.2	426±15	9.1±0.2	158.79	5.11±0.06	163.90	163.15
U12-H3	fixed*	fixed*	fixed*			12.19	1.81±0.01	14.00	13.98
U15-N3	18.54±1.74	361±56	10.1±1.4			161.15	-3.88±0.25	157.27	N/A
U15-H3	fixed*	fixed*	fixed*			13.94	-2.14±0.01	11.80	N/A
G22-N1	14.32±0.30	307±115	9.2±3.5			148.41	-1.12±0.06	147.29	147.56
G22-H1	fixed*	fixed*	fixed*			12.79	0.00±0.00	12.79	12.92
G23-N1	18.97±0.55	425±12	9.1±0.2			142.92	5.07±0.08	147.99	147.58
G23-H1	fixed*	fixed*	fixed*			10.61	1.85±0.01	12.46	12.58

* R_2 can not be well defined by fitting the CEST profile, and thus was fixed to a typical value (25 s^{-1}). The parameters of H1/H3 k_{ex} and p_B were fixed to the globally fitting results.

Supplementary Table 11. Acquisition parameters and fitting results of RD experiments for T3 RNA. The upper table shows the spin-lock powers ($\omega_{SL}/2\pi$) and offsets ($\Omega/2\pi$) used in the ^{15}N $R_{1\rho}$ experiment at varying temperatures. The lower table lists the exchange parameters obtained by individually and globally fitting ^{15}N $R_{1\rho}$ and ^{1}H^{N} CEST data to a two-state model, as well as the experimental and predicted chemical shifts of ES at 10 °C.

Residue	T (°C)	$\omega_{SL}/2\pi$ [Hz] & {$\Omega/2\pi$ [Hz]}
U13-N3<br>U23-N3	10.0	100 & {-400,-350,-300,-250,±200,±150,±100,±50}<br>200 & {-500,±400,-350,-300,-250,±200,-150,±100,-75,±50}<br>300 & {-700,±500,-400,-350,±300,-250,-200,-150,±100,±50}
U15-N3	10.0	100 & {450,400,350,325,280,250,200,175,150,120,85,±50,-20,15}<br>200 & {500,±400,350,300,250,±200,150,±100,75,±50}<br>300 & {700,±500,400,350,±300,250,200,150,±100,±50}

Residue	R_2 (s^{-1})	k_{ex} (s^{-1})	p_B (%)	k_{ex} (s^{-1})	p_B (%)	$\bar{\omega}_{GS}$ [ppm]	$\Delta\bar{\omega}$ [ppm]	$\bar{\omega}_{ES}{}^{exptl}$ [ppm]	$\bar{\omega}_{ES}{}^{pred}$ [ppm]
U13-N3	14.61±0.23	1350±272	0.58±0.09	1435±179	0.50±0.06	162.21	-3.46±0.34	158.75	159.13
U13-H3	fixed*	fixed*	fixed*			13.68	-1.84±0.03	11.84	11.92
U15-N3	16.00±0.19	1459±279	0.48±0.13			158.47	3.09±0.30	161.56	N/A
U15-H3	fixed*	fixed*	fixed*			11.64	1.99±0.04	13.63	N/A
U23-N3	13.56±0.14	1443±209	0.48±0.06			162.51	-3.75±0.34	158.76	N/A
U23-H3	fixed*	fixed*	fixed*			13.79	-2.15±0.02	11.64	N/A

* R_2 can not be well defined by fitting the CEST profile, and thus was fixed to a typical value (25 s^{-1}). The parameters of H1/H3 k_{ex} and p_B were fixed to the globally fitting results.

Supplementary Table 12. Acquisition parameters and fitting results of RD experiments for T4 RNA. The upper table shows the spin-lock powers ($\omega_{SL}/2\pi$) and offsets ($\Omega/2\pi$) used in the ^{15}N $R_{1\rho}$ experiment at varying temperatures. The middle table lists the exchange parameters obtained by individually and globally fitting ^{15}N $R_{1\rho}$ and ^{1}H^{N} CEST data to a two-state model, as well as the experimental and predicted chemical shifts of ES at 10 °C. The lower table shows the exchange parameters of T4 RNA obtained from individual and global fit of ^{15}N $R_{1\rho}$ data at different temperatures.

Residue	*T* (°C)	$\omega_{SL}/2\pi$ [Hz] & {$\Omega/2\pi$ [Hz]}
G3-N1<br>U27-N3	10.0	100 & {500,450,400,375,350,325,300,275,250,200,150,±100,±50}
		200 & {650,550,500,450,400,350,325,300,275,250,200,±150,±100,±50}
		300 & {700,600,500,450,400,350,325,300,275,250,200,±150,100,±50}
		500 & {800,700,600,500,450,±400,350,300,250,±200,±150,100,±50}
G3-N1<br>U27-N3	5.0<br>&<br>15.0	100 & {400,375,350,325,300,275,250,225,200,150,±100,±50}
		200 & {650,550,500,450,400,350,325,300,275,250,200,±150,±100,±50}
		300 & {700,600,500,450,400,350,325,300,275,250,200,±150,100,±50}
		500 & {800,700,600,500,450,±400,350,300,250,±200,±150,100,±50}
U25-N3	10.0	100 & {-500,-450,-400,-375,-350,-325,-300,-275,-250,-200,-150,±100,±50}
		200 & {-650,-550,-500,-450,-400,-350,-325,-300,-275,-250,-200,±150,±100,±50}
		300 & {-700,-600,-500,-450,-400,-350,-325,-300,-275,-250,-200,±150,-100,±50}
		500 & {-800,-700,-600,-500,-450,±400,-350,-300,-250,±200,±150,-100,±50}

Residue	R_2 (s^{-1})	k_{ex} (s^{-1})	p_B (%)	k_{ex} (s^{-1})	p_B (%)	$\bar{\omega}_{GS}$ [ppm]	$\Delta\bar{\omega}$ [ppm]	$\bar{\omega}_{ES}{}^{exptl}$ [ppm]	$\bar{\omega}_{ES}{}^{pred}$ [ppm]
G3-N1	10.78±0.13	2075±135	0.59±0.04	1716±73	0.58±0.02	143.08	4.61±0.14	147.69	147.58
G3-H1	fixed*	fixed*	fixed*			10.61	1.87±0.01	12.48	12.58
U25-N3	10.19±0.18	1488±197	0.45±0.07			163.13	-3.69±0.19	159.44	N/A
U25-H3	fixed*	fixed*	fixed*			14.57	-2.40±0.02	12.17	N/A
U27-N3	10.71±0.08	1629±56	0.59±0.01			158.52	4.88±0.10	163.40	163.15
U27-H3	fixed*	fixed*	fixed*			11.95	1.98±0.01	13.93	13.98

* R_2 can not be well defined by fitting the CEST profile, and thus was fixed to a typical value (25 s^{-1}). The parameters of H1/H3 k_{ex} and p_B were fixed to the globally fitting results.

Residue	R_2 (s^{-1})	*T* (°C)	k_{ex} (s^{-1})	p_B (%)	k_{ex} (s^{-1})	p_B (%)	$\Delta\bar{\omega}$ [ppm]
G3-N1	12.36±0.10	5.0	1264±68	0.51±0.02	1157±66	0.48±0.01	4.71±0.13
U27-N3	12.07±0.08		1097±54	0.47±0.01			4.71±0.13
G3-N1	10.78±0.13	10.0	2075±135	0.59±0.04	1716±73	0.58±0.02	4.61±0.14
U27-N3	10.71±0.08		1629±56	0.59±0.01			4.88±0.10
G3-N1	9.27±0.13	15.0	3352±137	0.50±0.03	2752±104	0.62±0.03	5.12±0.22
U27-N3	9.46±0.13		2406±84	0.67±0.03			5.12±0.22

Supplementary Table 13. The B_1 fields and mixing times of the $^1H^N$ CEST experiment for each designed RNA.

RNA samples	B_1 fields (Hz)	Mixing times (ms)
**T1**	30	400
**T1-GAAA**	30	400
**T1-UUCG**	30	400
**T1-add1bp**	30	400
**T1-add2bp**	60	400
**T1-delAU**	30	400
**T2**	30	400
**T2-mirror**	60	400
**T3**	140	500
**T4**	90	400

Supplementary References

1. Parisien, M.; Major, F., The MC-Fold and MC-Sym pipeline infers RNA structure from sequence data. *Nature* **2008,** *452* (7183), 51-5.
2. Richardson, K. E.; Kirkpatrick, C. C.; Znosko, B. M., RNA CoSSMos 2.0: an improved searchable database of secondary structure motifs in RNA three-dimensional structures. *Database (Oxford)* **2020,** *2020*.
3. Zgarbova, M.; Otyepka, M.; Sponer, J.; Mladek, A.; Banas, P.; Cheatham, T. E., 3rd; Jurecka, P., Refinement of the Cornell et al. Nucleic Acids Force Field Based on Reference Quantum Chemical Calculations of Glycosidic Torsion Profiles. *J Chem Theory Comput* **2011,** *7* (9), 2886-2902.
4. Pierce, L. C.; Salomon-Ferrer, R.; Augusto, F. d. O. C.; McCammon, J. A.; Walker, R. C., Routine access to millisecond time scale events with accelerated molecular dynamics. *J Chem Theory Comput* **2012,** *8* (9), 2997-3002.
5. Humphrey, W.; Dalke, A.; Schulten, K., VMD: visual molecular dynamics. *J Mol Graph* **1996,** *14* (1), 33-8, 27-8.
6. Lu, X. J.; Bussemaker, H. J.; Olson, W. K., DSSR: an integrated software tool for dissecting the spatial structure of RNA. *Nucleic Acids Res* **2015,** *43* (21), e142.
7. Markley, J. L.; Ulrich, E. L.; Berman, H. M.; Henrick, K.; Nakamura, H.; Akutsu, H., BioMagResBank (BMRB) as a partner in the Worldwide Protein Data Bank (wwPDB): new policies affecting biomolecular NMR depositions. *Journal of Biomolecular NMR* **2008,** *40* (3), 153-5.
8. Lee, J.; Dethoff, E. A.; Al-Hashimi, H. M., Invisible RNA state dynamically couples distant motifs. *Proc Natl Acad Sci U S A* **2014,** *111* (26), 9485-90.
9. Dethoff, E. A.; Petzold, K.; Chugh, J.; Casiano-Negroni, A.; Al-Hashimi, H. M., Visualizing transient low-populated structures of RNA. *Nature* **2012,** *491* (7426), 724-8.
