# Supplementary material for: Rational design of hairpin RNA excited states reveals multi-step transitions
Source: Nat Commun. 2022 Mar 21;13:1523. doi: 10.1038/s41467-022-29194-8 (PMC8938425; doi:10.1038/s41467-022-29194-8)
Supplement: Supplementary file 3 — Description of Additional Supplementary Files [file 41467_2022_29194_MOESM3_ESM.pdf]

Description of Additional Supplementary Files

File Name: Supplementary Movie 1
Description: A typical process of ES-to-GS transition of T4 RNA (top to bottom).

File Name: Supplementary Movie 2
Description: A typical process of ES-to-GS transition of T4 RNA (bottom to top).

File Name: Supplementary Movie 3
Description: A typical process of ES-to-GS transition of T1 RNA (top to bottom).

File Name: Supplementary Movie 4
Description: A typical process of GS-to-ES transition of T1 RNA (bottom to top).

File Name: Supplementary Movie 5
Description: A typical process of ES-to-GS transition of T2 RNA (top to bottom).
