# Supplementary material for: Rational design of hairpin RNA excited states reveals multi-step transitions
Source: Nat Commun. 2022 Mar 21;13:1523. doi: 10.1038/s41467-022-29194-8 (PMC8938425; doi:10.1038/s41467-022-29194-8)
Supplement: Supplementary file 9 — Reporting Summary [file 41467_2022_29194_MOESM9_ESM.pdf]

nature portfolio

Corresponding author(s): Yi Xue

Last updated by author(s): Dec 28, 2021

Reporting Summary

Nature Portfolio wishes to improve the reproducibility of the work that we publish. This form provides structure for consistency and transparency in reporting. For further information on Nature Portfolio policies, see our Editorial Policies and the Editorial Policy Checklist.

Statistics

For all statistical analyses, confirm that the following items are present in the figure legend, table legend, main text, or Methods section.

n/a	Confirmed	
☐	☒	The exact sample size (*n*) for each experimental group/condition, given as a discrete number and unit of measurement
☐	☒	A statement on whether measurements were taken from distinct samples or whether the same sample was measured repeatedly
☒	☐	The statistical test(s) used AND whether they are one- or two-sided<br>*Only common tests should be described solely by name; describe more complex techniques in the Methods section.*
☒	☐	A description of all covariates tested
☒	☐	A description of any assumptions or corrections, such as tests of normality and adjustment for multiple comparisons
☐	☒	A full description of the statistical parameters including central tendency (e.g. means) or other basic estimates (e.g. regression coefficient) AND variation (e.g. standard deviation) or associated estimates of uncertainty (e.g. confidence intervals)
☒	☐	For null hypothesis testing, the test statistic (e.g. *F*, *t*, *r*) with confidence intervals, effect sizes, degrees of freedom and *P* value noted<br>*Give P values as exact values whenever suitable.*
☒	☐	For Bayesian analysis, information on the choice of priors and Markov chain Monte Carlo settings
☒	☐	For hierarchical and complex designs, identification of the appropriate level for tests and full reporting of outcomes
☒	☐	Estimates of effect sizes (e.g. Cohen's *d*, Pearson's *r*), indicating how they were calculated

Our web collection on statistics for biologists contains articles on many of the points above.

Software and code

Policy information about availability of computer code

Data collection	Bruker Topspin 3.2 (600MHz NMR spectrometer), Bruker Topspin 3.5 (800MHz NMR spectrometer)
Data analysis	NMRPipe 9.6, Sparky 3.113, Amber 18, VMD 1.9.4, PyMOL 1.8.6.2, custom python scripts (Python 2.7.12, NumPy 1.11.0, Matplotlib 1.5.1), DSSR 1.5.1, ChemEx 0.6.1. The code for rational design and kinetic simulations are available at https://github.com/snowrecall/RNA-design.

For manuscripts utilizing custom algorithms or software that are central to the research but not yet described in published literature, software must be made available to editors and reviewers. We strongly encourage code deposition in a community repository (e.g. GitHub). See the Nature Portfolio guidelines for submitting code & software for further information.

Data

Policy information about availability of data

All manuscripts must include a data availability statement. This statement should provide the following information, where applicable:

- Accession codes, unique identifiers, or web links for publicly available datasets
- A description of any restrictions on data availability
- For clinical datasets or third party data, please ensure that the statement adheres to our policy

All data have been provided in the main text or the Supplementary Information, including sequence information of RNA constructs that we measured, the protocol and associated parameters of running MD simulations, NMR spectra with resonance assignments, the acquisition parameters and fitting results of NMR relaxation dispersion experiments, and MD movies. The 1HN-15N resonance assignments of the designed RNAs have been deposited in the BMRB database under accession numbers 51238 and 51241-51249. Other data that support the findings of this study, if any, are available from the corresponding author upon reasonable request.

Field-specific reporting

Please select the one below that is the best fit for your research. If you are not sure, read the appropriate sections before making your selection.

☒ Life sciences ☐ Behavioural & social sciences ☐ Ecological, evolutionary & environmental sciences

For a reference copy of the document with all sections, see nature.com/documents/nr-reporting-summary-flat.pdf

Life sciences study design

All studies must disclose on these points even when the disclosure is negative.

Sample size	For each designed hairpin RNA, two samples (unlabeled and uniformly 13C/15N-labeled) were prepared, and they showed almost identical imino 2D spectra. The first sample was used for the resonance assignment, while the second was used for the NMR relaxation dispersion measurement.
Data exclusions	No data were excluded from the analyses.
Replication	The NMR relaxation dispersion experiments were performed at least twice times to ensure the reproducibility. The first run of the experiment used two time delays to measure relaxation rates, while the final run of the experiment used six time delays (and sometimes a more concentrated sample) to measure relaxation rates. The MD simulations were repeated many times in order to obtain adequate statistics (T4 MD was repeated for 94 times, T1 for 161 times, and T2 for 118 times; the details have been described in the manuscript). All attempts at replication were successful.
Randomization	No relevant statistical analyses have been conducted in this work, as randomization design is not applicable for NMR experiments or MD simulations.
Blinding	No relevant statistical analyses have been conducted in this work, as blinding design is not applicable for NMR experiments or MD simulations.

Reporting for specific materials, systems and methods

We require information from authors about some types of materials, experimental systems and methods used in many studies. Here, indicate whether each material, system or method listed is relevant to your study. If you are not sure if a list item applies to your research, read the appropriate section before selecting a response.

Materials & experimental systems

n/a	Involved in the study	
☒	☐	Antibodies
☒	☐	Eukaryotic cell lines
☒	☐	Palaeontology and archaeology
☒	☐	Animals and other organisms
☒	☐	Human research participants
☒	☐	Clinical data
☒	☐	Dual use research of concern

Methods

n/a	Involved in the study	
☒	☐	ChIP-seq
☒	☐	Flow cytometry
☒	☐	MRI-based neuroimaging
